# Benchmarking enrichment analysis methods with the disease pathway network

**DOI:** 10.1093/bib/bbae069

**Published:** 2024-03-02

**Authors:** Davide Buzzao, Miguel Castresana-Aguirre, Dimitri Guala, Erik L L Sonnhammer

**Affiliations:** Department of Biochemistry and Biophysics, Stockholm University, Science for Life Laboratory, Box 1031, 171 21 Solna, Sweden; K7 Department of Oncology-Pathology, Karolinska Institute, 171 77 Stockholm, Sweden; Department of Biochemistry and Biophysics, Stockholm University, Science for Life Laboratory, Box 1031, 171 21 Solna, Sweden; Department of Biochemistry and Biophysics, Stockholm University, Science for Life Laboratory, Box 1031, 171 21 Solna, Sweden

**Keywords:** systems biology, pathway enrichment analysis, functional enrichment, gene set enrichment analysis, disease pathway network, gene expression data

## Abstract

Enrichment analysis (EA) is a common approach to gain functional insights from genome-scale experiments. As a consequence, a large number of EA methods have been developed, yet it is unclear from previous studies which method is the best for a given dataset. The main issues with previous benchmarks include the complexity of correctly assigning true pathways to a test dataset, and lack of generality of the evaluation metrics, for which the rank of a single target pathway is commonly used. We here provide a generalized EA benchmark and apply it to the most widely used EA methods, representing all four categories of current approaches. The benchmark employs a new set of 82 curated gene expression datasets from DNA microarray and RNA-Seq experiments for 26 diseases, of which only 13 are cancers. In order to address the shortcomings of the single target pathway approach and to enhance the sensitivity evaluation, we present the Disease Pathway Network, in which related Kyoto Encyclopedia of Genes and Genomes pathways are linked. We introduce a novel approach to evaluate pathway EA by combining sensitivity and specificity to provide a balanced evaluation of EA methods. This approach identifies Network Enrichment Analysis methods as the overall top performers compared with overlap-based methods. By using randomized gene expression datasets, we explore the null hypothesis bias of each method, revealing that most of them produce skewed *P*-values.

## INTRODUCTION

Enrichment analysis (EA) is a popular approach for gaining biological insights from high-throughput experiments. Gene expression profiles from DNA microarray or RNA-Seq research are commonly used as input data to EA. In a typical experiment, expression profiles for thousands of genes are obtained from a collection of samples from two categories such as case/control or treated/untreated, from which differentially expressed genes (DEGs) can be extracted. The goal of EA is to condense these profiles or DEG lists into a concise set of impacted biological functions or processes that can provide a systems level view of the changes and lead researchers to disease biomarkers or therapeutic targets. Functional gene sets representing molecular functions and biological processes are defined, e.g. by the Gene Ontology (GO) [1], pathway databases such as Kyoto Encyclopedia of Genes and Genomes (KEGG) [2], Reactome [3], or experimentally derived gene set databases such as DisGeNET [4] or MSigDB [5].

EA methods can be grouped into four categories according to their procedural framework [6] that also mirrors the timeline of their development: (i) overlap-based methods test the significance of DEGs that overlap a functional gene set, (ii) per-gene scoring methods look for enriched ranking of a functional gene set in the sorted list of gene expressions, (iii) pathway topology methods acquire topological information from databases such as KEGG or Reactome to weigh the importance of each gene in the tested pathway and (iv) network-based methods look for enrichment of network links between DEGs and a functional gene set.

Despite the availability of a large range of EA methods, it is unclear which ones are the best for a given dataset. Various methods have been developed claiming to be better than DAVID [7] and GSEA [8], yet these remain highly cited methods for EA. However, many such benchmarks were based on simulated data. Tarca *et al.* [9] pioneered biological data benchmarking by using experimental datasets from diseases that could be associated with a particular pathway. Several recent enrichment evaluation studies have adopted this technique [10–13], yet the Tarca *et al.* benchmark has some problems such as only containing 19 diseases, 12 of which are cancer-related. Furthermore, to calculate sensitivity, the target pathway is treated as the single true positive and all other pathways are assumed to be false positives. The shortcoming of the single target pathway approach was first addressed by using mouse knockout experiments (KO) with a known KO gene, by considering all pathways that contain the KO gene as positive, and the rest as negative pathways [12]. However, this is only possible for a small number of datasets, and the validity of the positive and negative assignments can be questioned. Geistlinger *et al.* [13] updated the Tarca benchmark by including RNA-Seq data and a new scoring metric that uses MalaCards [14] to associate a disease dataset with more than one pathway. MalaCards scores disease relevance for a gene based on literature co-citation and experimental data and summarizes per-gene relevance across the GO and KEGG gene sets. The benchmark calculates a ‘phenotype relevance’ score based on the EA rankings and the precompiled relevance rankings. The approach is only based on relevance scoring and does not penalize false negatives, which is also the case for the Tarca benchmark. Because of this, the results tend to be biased to give an unfair advantage to methods that have low sensitivity but high specificity. Obtaining a good balance between these metrics is very important for a general benchmark.

**Figure 1:**
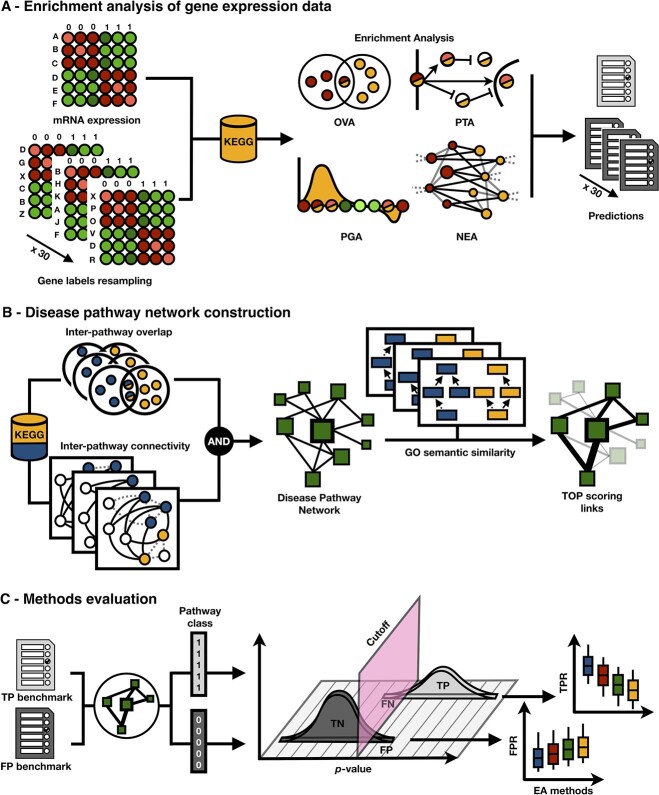
Procedural framework of the enrichment analysis benchmark. (**A**) EA methods are divided into four categories: OVA, PGA, PTA and NEA, that differ in terms of the data and extra resources required for their input. All methods were run on 82 experimental expression datasets and on 2460 random datasets. (**B**) The KEGG pathway database is used to evaluate the EA methods. Each dataset is paired with the pathway that matches the disease and other pathways that are significantly related to the target pathway, as well as being top ranked by GO semantic similarity. Related pathways form the Disease Pathway Network which is based on both significant overlap and network connectivity. (**C**) Sensitivity (TPR) and specificity (1-FPR) are calculated for each method from their predictions on experimental and random datasets, respectively.

The aim of this study is to present a generalized benchmark of the most popular EA methods, representing all four categories of existing approaches. As illustrated in Figure 1, the benchmark is structured in three parts. First, we provide a new collection of 76 curated DNA-microarray and 6 RNA-Seq gene expression datasets for 26 diseases, only 13 of which are neoplastic conditions. We ran 14 EA methods (two overlap-based, six per-gene scoring, four network-based and two topology-based) on the gene expression data against KEGG pathways. Second, we introduce the Disease Pathway Network to counteract the shortcoming of the single target pathway approach and improve the sensitivity assessment in an unbiased way. Third, we measure the sensitivity and specificity of the methods in an independent and balanced manner. Focusing on the performance under the null hypothesis, we further assess potential biases of each method and pathway.

## MATERIALS AND METHODS

### Pathways database

KEGG is a popular collection of 16 databases that contain genomic information, biological pathways, disease- and drug-related data. We used the R package KEGGREST (v.1.34.0) to retrieve 318 KEGG (Release 101.0+/02–20, Feb 22) human pathways with at least 15 associated genes, which corresponds to the size of 95% of all the pathways, and 92 of these pathways are related to human diseases and belong to 10 different subclasses, i.e. Endocrine and metabolic disease, Neurodegenerative disease, Substance dependence, Infectious disease: bacterial, Infectious disease: parasitic, Infectious disease: viral, Cancer: overview, Cancer: specific types, Immune disease and Cardiovascular disease.

### Expression data

By querying the Gemma resource [15] using the 92 KEGG disease pathway names, we retrieved 82 datasets via the Gemma.R package (v0.99.1). Only datasets with a case–control observational study design, originally deposited in GEO [16], were included. Datasets were either of high quality during submission to GEO or have undergone careful data and annotation curation in Gemma, e.g. by flagging or removing outlier samples. In order to guarantee a high quality of gene expression, we required (i) a minimum number of three samples per condition (case or control), (ii) no drug treatment in the study, (iii) batch effect either not detected or corrected and (iv) a minimum of 15 DEGs at an FDR-corrected *P*-value of less than 0.2. DNA-microarray expression was quantile normalized and log-transformed. RNA-Seq experiments were retrieved as logarithm of counts per million reads (log_2_CPM). Probes were mapped to Gene IDs using the annotation table provided by Gemma, when multiple probes mapped to the same gene they were averaged. Basic analyses such as (co-)expression distribution plots, PCA analysis and differential expression analysis with volcano plots were performed to confirm the quality of each of the dataset, and reported as Supplementary Materials (see Supplementary Data available online at http://bib.oxfordjournals.org/). For the ‘ulcerative colitis’ datasets, there was no exact match. Therefore, we used genes associated with ‘inflammatory bowel disease’ of which ‘ulcerative colitis’ is a subtype. A detailed description of each dataset is shown in Supplementary Table 1 (see Supplementary Data available online at http://bib.oxfordjournals.org/).

All datasets were considered to have an unpaired design. Limma (v3.54.0) was used to extract gene-wise moderated *t*-test scores of expression between case and control samples, run with trend = True for RNA-Seq datasets. DEGs were defined as genes with Benjamini–Hochberg FDR-corrected *P*-values of less than 0.1 for 72 datasets, and of less than 0.2 for 10 datasets. When the number of DEGs exceeded 500, only the 500 most significant DEGs were selected, and 500 was chosen because KEGG pathways are mostly not larger, and although the choice of cutoff has a relatively small impact, using the top 500 DEGs was found to give a close to optimal performance for different methods (Supplementary Figure 1, see Supplementary Data available online at http://bib.oxfordjournals.org/).

### Disease pathway network construction

#### Inter-pathway connectivity

HumanNet-XC is a functional network of human genes that comprises inferred associations of gene co-expression, PPIs, genetic interactions, protein domain co-occurrence and genomic context similarity from experimental datasets [17]. It was shown to have the highest protein coverage of all tested integrated human gene networks [17]. HumanNet shares gene ID nomenclature with KEGG meaning that its use also eliminated the need to accommodate the network’s gene vocabulary to pathways, which minimized inherent translation issues.

Inter-pathway connectivity *IPC(A,B)* was computed as the sum of the number of direct links *DL(A,B)* and shared neighbors *SN(A,B)* between the genes (e.g. *a* ∈ *A*, *b* ∈ *B*) of any two pathways (e.g. *A, B*), as connected in HumanNet-XC (v3) with 20% top scoring links (Eq. 1)


(1) \begin{equation*} IPC\left(A,B\right)= DL\left(A,B\right)+ SN\left(A,B\right). \end{equation*}

The number of direct links *DL(A,B)* was computed as the number of links between *A* and *B*.

The number of shared neighbors *SN(A,B)* was computed as the number of genes that were not part of pathways *A* and *B* yet were connected to at least one gene in both *A* and *B*.

The *P*-value of the connectivity between two pathways was tested with a degree-aware subsampling test of 1000 random samples, i.e. for the *A* against *B* test, *A* remained unchanged and *B* was replaced by a gene set where each gene was randomly sampled from genes with similar node degree in the network. This was achieved by sorting the 7248 KEGG genes by node degree and grouping them into bins of 100 genes. The binning made it possible to sample high-degree nodes that do not have a substitute with exactly the same degree. The *P*-value may be different when sampling *A* and *B*, for instance due to different sizes or degree distributions, hence we performed the test in both directions and combined the two *P*-values for each pathway pair using Fisher’s method [18].

### Inter-pathway overlap

To calculate the significance of the gene overlap between two pathways, the same bi-directional degree-aware subsampling method as above was used, with 1000 random samples. Here, the overlap was expressed as Jaccard index *J(A,B)* (Eq. 2)


(2) \begin{equation*} J\left(A,B\right)=\frac{\left|A\cap B\right|}{\left|A\cup B\right|} \end{equation*}

### GO semantic similarity

For any two pathways that passed the overlap and connectivity tests, the semantic similarity of GO keywords was used as a proxy for their functional similarity. In particular, to compute similarity scores, we used the Wang *et al.* graph-based method [19] which uses the topology of the GO Directed Acyclic Graph structures as implemented in the function clusterSim from the R package GOSemSim (v2.20.0). See Supplementary Materials (see Supplementary Data available online at http://bib.oxfordjournals.org/) for a more detailed description.

The network was built by assembling edges that passed both the inter-pathway overlap and connectivity tests with an FDR-corrected *P*-value below 0.05. Edge weights were then added by measuring the GO semantic similarity for the pathway–pathway relationship that the edge represents.

### Performance measures

In this study, the positive and negative benchmarks were independent. In the positive benchmark, true positives (TP) and false negatives (FN) were positive pathways (i.e. target or target-related) that were identified as significantly related (*P*-value < 0.05) and not significantly related (*P*-value ≥ 0.05), respectively. Similarly, in the negative benchmark, true negatives (TN) and false positives (FP) were negatives (i.e. unrelated pathways) that were identified as non-significant or significant, respectively. The negative benchmark was designed by randomly sampling gene labels from the whole genome for the 82 datasets and repeating the procedure 30 times. Methods taking DEGs as input are inapplicable when only a few or nearly all genes are differentially expressed, but by utilizing gene label resampling, we get the same number of DEGs per dataset as originally and could estimate false positive rate (FPR) over 2460 × 318 tests for each method. To counteract the imbalance between positive and negative pathways, we considered only the number of target and target-related pathways from the positive benchmark to compute TN and FP in the negative benchmark. Having the definition of TP, TN, FP and FN, we extracted true positive rate (TPR, or sensitivity) and true negative rate (TNR, or specificity, or 1-FPR) as follows:


(3) \begin{equation*} \mathrm{TPR}=\frac{\mathrm{TP}}{\mathrm{TP}+\mathrm{FN}} \end{equation*}

(4) \begin{equation*} \mathrm{TNR}=\frac{\mathrm{TN}}{\mathrm{TN}+\mathrm{FP}} \end{equation*}

and used the geometric mean of TPR and TNR (G-mean) to condense the method performances in one balanced, comprehensive and robust summary index (Eq. 5)


(5) \begin{equation*} \mathrm{G}-\mathrm{mean}=\sqrt{\mathrm{TPR}\times \mathrm{TNR}}\; . \end{equation*}

To underline the importance of collecting TP pathways among the top predictions, we also extracted the median relative rank. A relative rank is the fraction of ranks more significant than the TP pathway’s rank in the predictions after sorting by *P-*value. In the presence of ties, we averaged the ranks of the tied pathways.

### Enrichment analysis methods

The EA methods selected for the benchmark are listed in Table 1. The methods under investigation belong to four different categories: (i) OVerlap Analysis (OVA), Per-Gene score Analysis (PGA), Pathway Topology Analysis (PTA) and Network Enrichment Analysis (NEA) methods. We summarize here the main characteristics of each category and provide a short description of each method, see Supplementary Material (see Supplementary Data available online at http://bib.oxfordjournals.org/) for more details.

**Table 1:** Enrichment analysis methods under benchmark

Method	Category	Gene ranking statistic	Enrichment scoring	Significance estimation	R-function / package	Author
EASE	OVA	–	overlap	mod. Fisher’s exact test	fisher.test	[20]
Fisher	OVA	–	overlap	Fisher’s exact test	fisher.test	–
CAMERA	PGA	*t*_MOD_	*t*_IGC_	two-sample *t*-test	limma (v3.50.1)	[21]
fGSEA	PGA	*t*_SNR_	KS-like statistic	gene set permutations	fgsea (v1.20.0)	[22]
GSEA	PGA	*t*_SNR_	KS-like statistic	sample permutations	DOSE (v3.20.0)	[8]
GSA	PGA	*t*_SAM_	maxmean	gene + sample permutations	gsa (v1.03.2)	[23]
GSVA	PGA	–	KS-lke statistic	moderated *t*-test	gsva (v1.42.0)	[24]
PADOG	PGA	|*t*_MOD_|	weighted mean	sample permutations	padog (v1.36.0)	[25]
ROAST	PGA	*t*_MOD_	weighted mean	rotation	limma (v3.50.1)	[26]
CePa-ORA	PTA	–	overlap + centrality-based score	sample permutations	CePa (v0.8.0)	[27]
SPIA	PTA	–	overlap + perturbation-based score	sample permutations	spia (v2.46.0)	[28]
ANUBIX	NEA	–	crosstalk	degree-aware DEGs sampling + beta binomial test	ANUBIX (v1.0.3)	[29]
BinoX	NEA	–	crosstalk	network randomization + binomial test	BinoX (v0.9.6)	[30]
NEAT	NEA	–	crosstalk	hypergeometric test	neat (v1.2.3)	[31]
netPEA	NEA	–	mean RWR flow	DEGs sampling + *z-*test	dnet (v1.1.7)	[32]

PTA and NEA methods take DEGs as input. PGA methods perform gene statistics via adaptations of Student’s *t*-test: *t*_MOD_ is moderated *t-*test, *t*_SNR_ is *t-*like signal-to-noise ratio, *t*_SAM_ is SAM’s *t* accounting for small variability at low expression values. All methods were run in an R (v4.1.0) version-controlled conda environment using the original packages, with the exception of EASE, Fisher and netPEA that were run using our own R implementations, and BinoX that is a C++ program.

OVA methods test the proportion of DEGs in a functional gene set against a discrete probability distribution model followed by the extraction of one-sided *P*-values. Fisher tests the overlap of DEGs in a pathway using Fisher’s exact test. EASE [20] employs a conservative modification of Fisher’s exact test by subtracting 1 from the overlap of DEGs.

PGA methods work with each gene separately, employing a statistical model to connect the response to the expression of each gene. In most cases, each gene undergoes a local statistical test that is used to determine a parametric or permutation-based *P*-value of variation of expression. A global statistical analysis is then performed to assign an enrichment score (ES) to a functional set of genes. CAMERA [21] accounts for gene-to-gene correlations to adjust the gene set statistic. GSEA [8] evaluates whether the distribution of gene ranks (e.g. fold changes or *P*-values) in the gene set differs from a uniform distribution using a Kolmogorov–Smirnov (KS) such as statistic, and tests the ES via sample permutations. fGSEA [22] efficiently conducts pre-ranked GSEA, estimating *P*-values through permutation of gene sets of the same size. GSA [23] uses the maxmean statistic and tests the ES via gene and sample permutations. GSVA [24] also uses a KS-like statistics test but applies it to normalized expression data to calculate pathway ESs for each sample in the dataset individually. *P*-values are derived for each pathway by testing ES differences between the control and case samples via a moderated *t*-test. PADOG [25] pre-computes gene frequencies in the pathway database, calculates the ES weighted by gene frequencies and tests the ES via sample permutations. ROAST [26] implements a self-contained test to estimate pathway *P*-values using rotation, a Monte Carlo simulation technique. For permutation-based methods, the significance of the global statistics for each pathway was evaluated using 2000 iterations. Other settings are kept to default values, with the exception of CAMERA that was run in two configurations: (i) CAMERA_fixed accounting for fixed inter-gene correlation of 0.01 and (ii) CAMERA_flex extracting dataset-specific inter-gene correlation.

PTA methods acquire topological information from databases such as KEGG or Reactome to weight the importance of each gene in the tested pathway. PTA methods extract a per pathway score and perform size-aware permutations to extract *P*-values of enrichment. CePa-ORA [27] measures centrality metrics on gene networks to extract node weights and calculates the pathway ES by summing the weights of DEGs, with significance determined through gene permutations. SPIA [28] assesses the perturbation of a pathway by propagating fold changes through a signed directed graph representation of the pathway topology. It utilizes activation and inhibition weights, comparing observed net perturbation to a randomized scenario.

NEA methods evaluate the interconnectivity between DEGs and functional gene sets in the context of a functional association network, where a parametric or permutation-based approach is used to assess statistical significance of the measured interconnectivity. ANUBIX [29], BinoX [30] and NEAT [31] find network crosstalks between DEGs and a pathway. ANUBIX tests the crosstalk significance against a beta-binomial distribution, adapted for each pathway and DEG set and estimated through a degree-aware resampling procedure. BinoX compares the crosstalk to a binomial null distribution based on randomization of the network. NEAT tests the crosstalk significance against a hypergeometric null distribution. If the expected crosstalk is larger than the observed, both BinoX and NEAT predict depletion by testing the lower tail of the distribution. As depletion is not considered in the benchmark, we took 1 − *P* value as the significance of enrichment for depleted gene sets. netPEA [32] employs Random Walk with Restarts (RWR) on a network using DEGs as seed nodes and then averages the probabilities at steady state to extract a pathway ES and compares that to a standardized normal distribution based on DEGs resampling. FunCoup [33] and STRING [34] were used as underlying networks with a default confidence link score above 0.8 and 800, respectively.

## RESULTS

### The disease pathway network for generalized benchmarking

**Figure 2:**
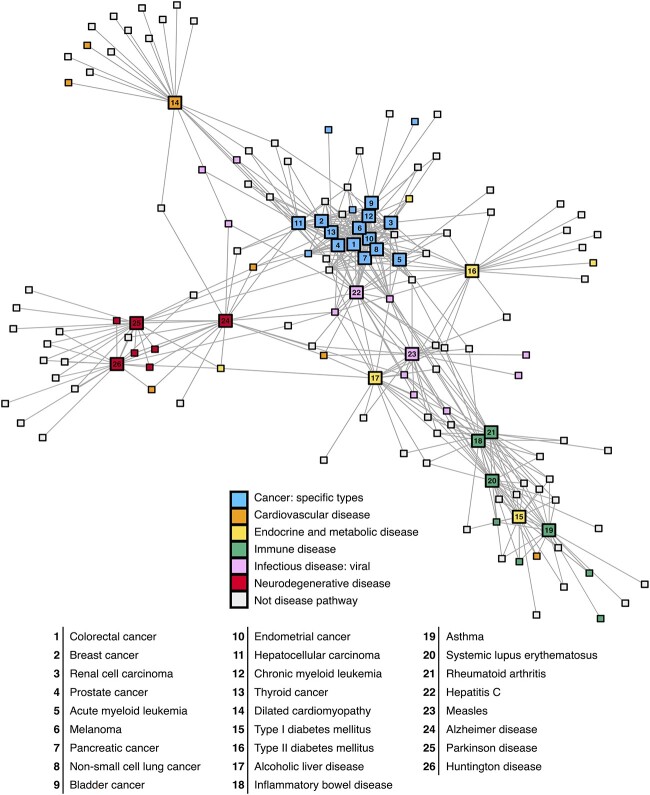
The Disease Pathway Network in the benchmark. An overview of KEGG diseases and their top 20 connected pathways, highlighting subclasses included in the benchmark. Target disease pathways are depicted with a larger node size and are labeled with an internal node id. Links between non-target pathways are not shown as they are out of the scope of this benchmark. The colors correspond to the KEGG disease subclass a pathway belongs to. The width of the links represents the GO semantic similarity, which ranges from 0.77 to 0.98 in this network. The network was visualized using R iGraph (v1.3.0).

To achieve high sensitivity in the benchmark, we created the Disease Pathway Network that connects target pathways to other related pathways. It was produced by first extracting 92 pathways from KEGG that are associated with human diseases, and connecting these to other KEGG pathways based on significant gene overlap and network connectivity. To rank these pathway−pathway relations in a functionally relevant way, we scored them using GO semantic similarity as described in the Supplementary Materials (see Supplementary Data available online at http://bib.oxfordjournals.org/). To ensure an equal representation of the benchmarked disease pathway subnetworks, we restricted them to the top 20 linked pathways for each target disease pathway. This decision was motivated by the limited number of total associations observed in Parkinson’s and Huntington’s diseases (Supplementary Table 2, see Supplementary Data available online at http://bib.oxfordjournals.org/). This resulted in a Disease Pathway Network of 212 pathways and 1520 links in total. Figure 2 shows a subnetwork of the target disease pathways in the Disease Pathway Network, with 143 pathways and 462 links. It illustrates the biological coherence of our disease pathway model, with stronger connections observed between diseases within the same class.

Some pathways are very central in the Disease Pathway Network, with ‘Hepatitis C’ having the largest number of connections. In the broader network, viral, parasitic and bacterial infections emerge as the most connected class (Supplementary Figure 2, see Supplementary Data available online at http://bib.oxfordjournals.org/), with Hepatitis C ranking third with 46 links, following Hepatitis B (54 links) and Yersinia infection (47 links). Viral infections, including Hepatitis C, often involve complex interactions with host pathways, influencing a range of cellular processes [35]. This can explain the observed centrality of Hepatitis C within the Disease Pathway Network. Our dataset is linked to 26 target diseases, and because disease representation varies, certain pathways were tested more often with our 82 benchmark datasets (Supplementary Figure 3, see Supplementary Data available online at http://bib.oxfordjournals.org/). The most frequently tested pathway was the ‘Neurotrophin signaling pathway’, which occurred in 61% of the tests, while at the other extreme, some pathways were only tested once.

We also calculated the Jaccard index between all disease pathways within the same KEGG subclass under ‘Human Diseases’ using the top 20 linked pathways (Supplementary Table 3, see Supplementary Data available online at http://bib.oxfordjournals.org/). Here, we observed that the ‘Cancer: specific types’ subclass of pathways represents a highly connected subnetwork, with an average Jaccard index of 0.45, ranging from a minimum of 0.18 (‘Glioma’–‘Small cell lung cancer’) to a maximum of 0.82 (‘Colorectal cancer’–‘Pancreatic cancer’). On the other hand, some pathway subclasses were not interconnected, such as ‘Endocrine and metabolic disease’ that includes type I and II diabetes. However, from a molecular mechanistic perspective, it makes more sense that type I diabetes, an autoimmune disease [36, 37], is connected to other autoimmune diseases such as Asthma rather than to type II diabetes. Another example of clustering across different disease classes is the ‘Hepatitis C’ pathway, under ‘Infectious disease: viral’, that instead shows strong associations with cancers. This may be explained by the fact that ‘Hepatitis C’ is a well-established risk factor for liver cirrhosis and liver cancer, despite primarily being a viral infection [38, 39].

### Benchmarking 14 EA methods of different types

**Figure 3:**
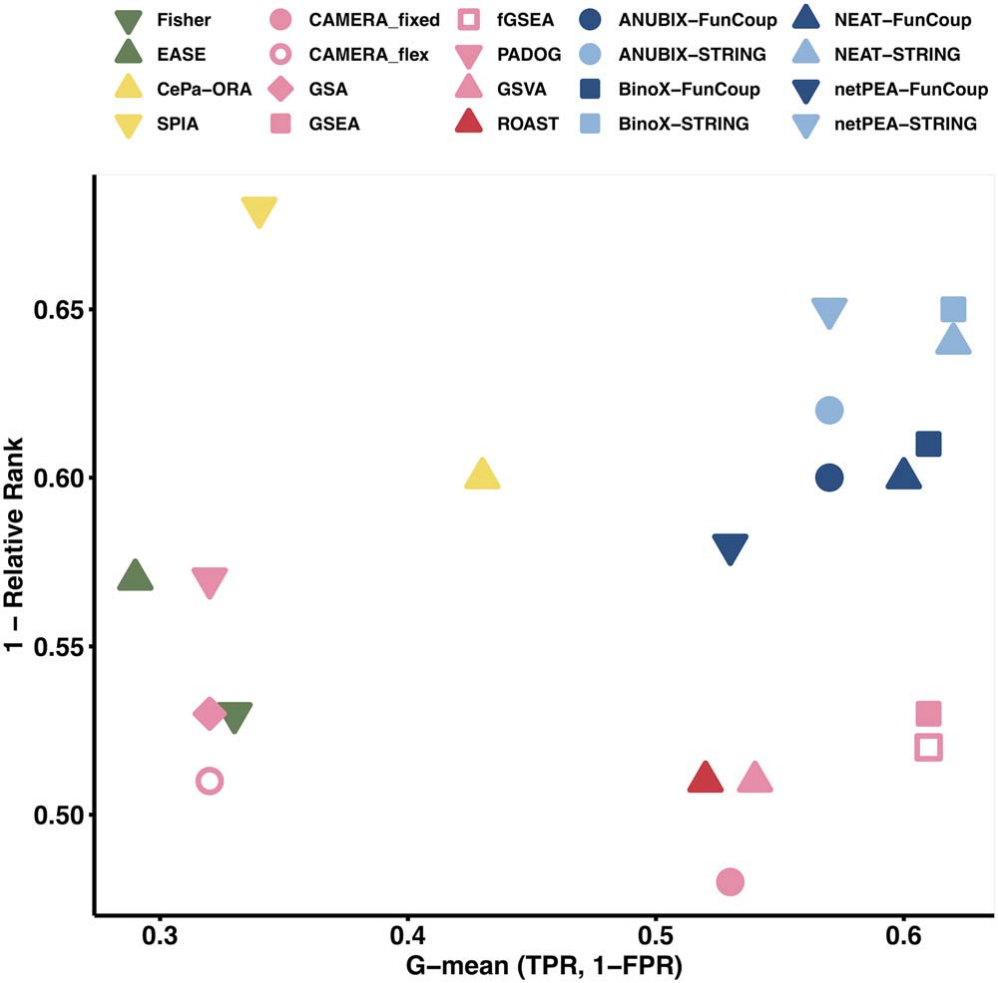
Summary of performance of 14 EA methods. G-mean of TPR and TNR (1-FPR) is shown versus 1-relative rank, using the top 20 linked pathways per target pathway in the Disease Pathway Network. An ideal method would rank the related pathways for a specific dataset at the top of enrichments and would assign significant *P*-values to them. In the figure, colors refer to EA categories: green for OVA, pink for PGA with light and dark shades for competitive and self-contained null hypothesis, yellow for PTA, and blue for NEA methods with light and dark shades for STRING-/FunCoup-based implementations.

We then set up a benchmark where the Disease Pathway Network was used to identify target-related pathways for each gene expression dataset based on its target pathway. Fourteen EA methods of all four categories of currently existing EA approaches were benchmarked by considering also the target-related pathways as true positives. The methods were compared in terms of relative pathway ranking, sensitivity and specificity. To avoid introducing a bias toward a particular functional association network, we ran the NEA methods with two different networks and refer to them with ^*^-FunCoup and ^*^-STRING in the method name. The performance of the investigated methods was summarized as a combination of average relative rank and G-mean of TPR and TNR (1 − FPR) using the top 20 linked pathways per disease pathway in the Disease Pathway Network (Figure 3). Supplementary Table 4 (see Supplementary Data available online at http://bib.oxfordjournals.org/) provides a comprehensive breakdown of scores, including the distinctions between True Positives (TP), True Negatives (TN), False Positives (FP) and False Negatives (FN).

The NEA methods exhibited the best combined performance, with BinoX and NEAT reaching the highest G-mean at 0.62, and BinoX scoring 0.65 at 1-Relative Rank. The PGA and PTA methods performed well in only one of the two scoring metrics. Among the PGA methods, fGSEA and GSEA had best G-mean at 0.61, while PADOG had the best ranking at 0.57. Although the PTA method SPIA achieved the highest rank performance of all methods, it is important to note that this was inflated by the limited number of pathways that PTA methods can test (see details about PTA methods in Supplementary Material, Supplementary Figure 4, see Supplementary Data available online at http://bib.oxfordjournals.org/). In our analysis, we assigned a *P*-value of 1 to all missing tests and computed the average rank for ties. A corollary of this is a low TPR, resulting in a poor G-mean. We noted that EASE got the lowest G-mean of 0.29, while CAMERA_fixed got the lowest rank performance at 0.48. A summary of the performance results with other cutoffs for the number of target-related pathways is shown in Supplementary Figure 5 (see Supplementary Data available online at http://bib.oxfordjournals.org/).

### Analysis of biases in the benchmarked methods

**Figure 4:**
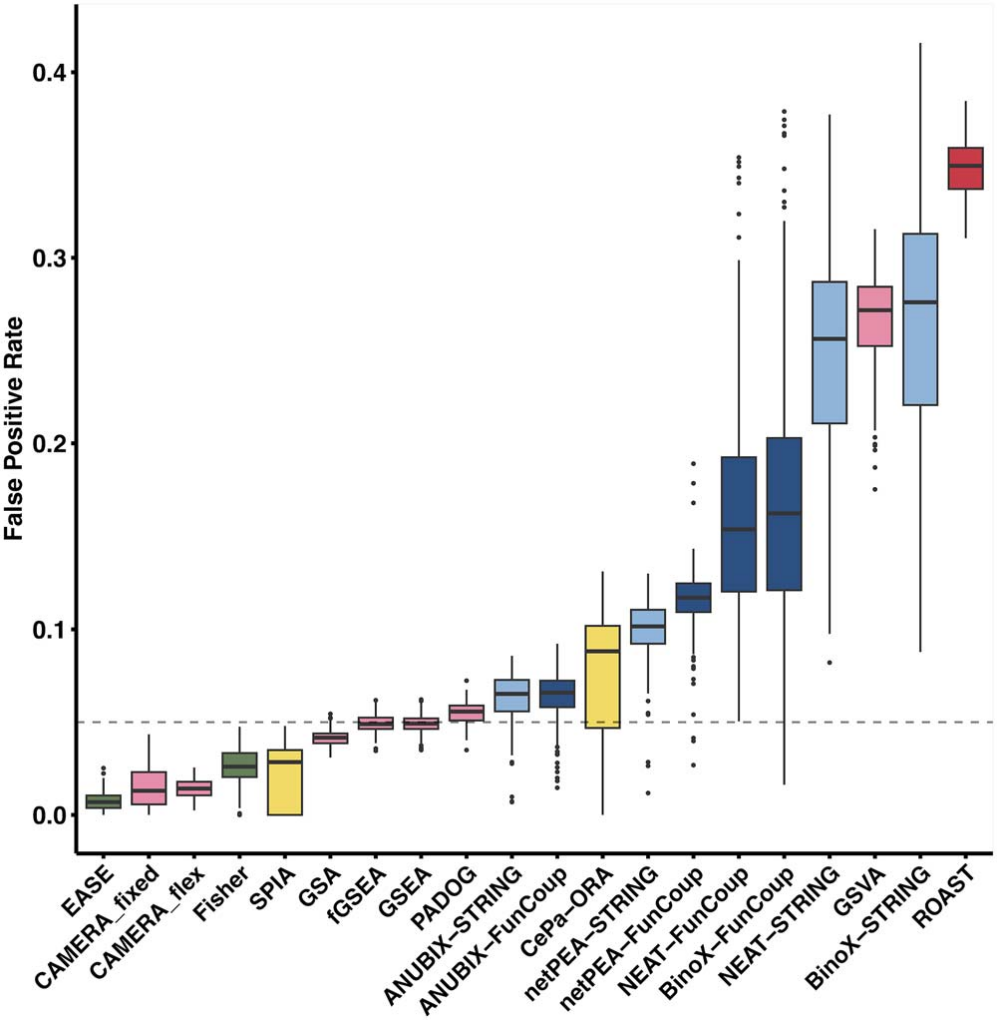
False positive benchmark. The FPRs of 318 KEGG pathways for all benchmarked EA methods. The data were generated by resampling from the genome to generate random gene labels for the positive benchmark datasets. The gray dashed line represents the requested significance level of 0.05. The box colors correspond to method categories: green for OVA, pink for PGA with light and dark shades for competitive and self-contained null hypothesis, yellow for PTA, and blue for NEA methods with light and dark shades for STRING-/FunCoup-based implementations.

We also evaluated the performance of the EA methods on randomized data (Figure 4). An ideal method would generate a uniform distribution of *P-*values from 0 to 1 across pathways when randomized data are used, with 5% of the *P-*values being lower than the cutoff at 0.05. Five of the methods were extremely conservative, with a median FPR below 3% and an FPR below the cutoff for all pathways. These include the OVA methods EASE and Fisher, the two CAMERA (PGA) methods and the PTA method SPIA. The other PTA method, CePA-ORA, was however non-conservative with a median FPR of 9%.

At the other end, four methods were extremely non-conservative with a median FPR above 16% and FPR above the cutoff for all pathways. These include the PGA methods ROAST and GSVA, and the NEA methods BinoX and NEAT. Both BinoX and NEAT were significantly more conservative when using FunCoup than STRING, with *P* = 1.6e−53 and *P* = 4.4e−55, respectively, in a Wilcoxon rank sum test. In contrast, the FPR for netPEA was significantly higher when using FunCoup than STRING (*P* = 1.9e−38). No significant difference between FunCoup and STRING was observed for ANUBIX (*P* = 5.2e−1). The methods GSA, fGSEA, GSEA, PADOG and ANUBIX performed very well in this test with a median FPR close to 5%.

**Figure 5:**
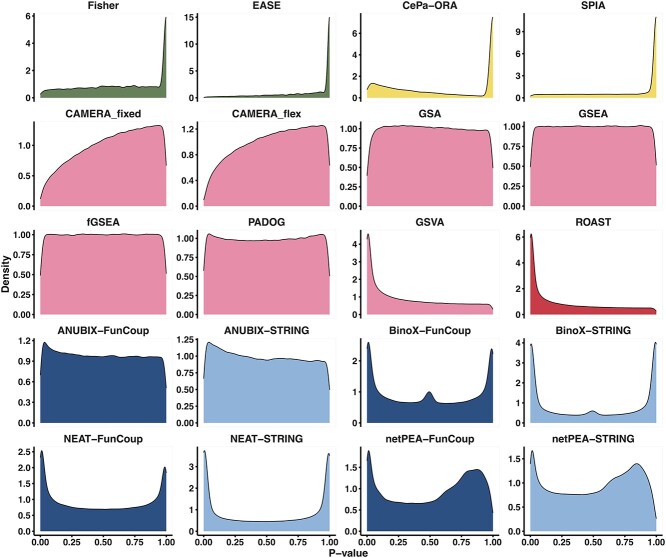
*P*-value distributions of all methods in the false positive benchmark. The *P*-values for all the pathways generated in the false positive benchmark are presented as density plots, depicting their distribution under the null hypothesis for each of the benchmarked methods. The ideal behavior is for the distribution to be uniform across the entire range from 0 to 1.

Under the null hypothesis, enrichment analysis methods often produce *P*-values that are either biased toward 0 or 1 or exhibit a bimodal distribution biased toward both extremes. Such a bias can affect the significance of the analysis, hence we examined the distribution of *P*-values for each method to see if it was right- or left-skewed (Figure 5). A right-skewed distribution (*P*-values biased toward 0) can potentially lead to false positives by reporting pathways as impacted when they are not. Conversely, a left-skewed distribution (*P*-values biased toward 1) can lead to false negatives by reporting pathways as not significant when they are actually impacted. GSEA was already reported to have no skewness bias [12] which we could confirm. Similarly, GSA, fGSEA and PADOG were unbiased in this benchmark. In contrast, the *P*-value distribution of the OVA methods, EASE and Fisher, was strongly skewed toward 1. At the other extreme, GSVA and ROAST had *P*-value distributions strongly skewed toward 0. BinoX, NEAT and netPEA had distinct bimodal *P*-value distributions, mainly due to wrong assumptions about the EA score distribution, while ANUBIX showed almost no skewness. The bimodal *P*-value distribution of the PTA methods is due to the high number of untested pathways. For the tested pathways, SPIA had no bias, whereas CePA-ORA was right-skewed for all of them.

The NEA methods, which performed the best overall, still exhibited biases for some of the pathways that we decided to analyze further. As NEA methods rely on functional association networks, we expected the bias to be related to the underlying network topology. Thus, for each pathway under study, we calculated a number of network properties and then measured how they correlated with FPR (Supplementary Figures 6 and 7, see Supplementary Data available online at http://bib.oxfordjournals.org/). We note a significant positive correlation for properties such as fraction of hubs, max degree and size with both FunCoup and STRING. With FunCoup only, there was a strong correlation with fraction of intralinks, for the methods BinoX and NEAT, which has been previously noted [29]. A high fraction of intralinks means that the pathway is isolated and exhibits community-like properties in the network.

Further support is provided by a significant positive Spearman correlation of 0.66 for BinoX and 0.71 for NEAT between the FPR and the fraction of intralinks when using FunCoup (Supplementary Figure 7, see Supplementary Data available online at http://bib.oxfordjournals.org/). BinoX and NEAT generalize the statistical properties of crosstalk for all pathways and are unable to adjust to unique properties of individual pathways, which are often highly non-random. In contrast, the correlation is weak in ANUBIX (0.06), which can be attributed to the fact that ANUBIX accurately predicts the expected degree of crosstalk by maintaining the pathways intact while performing a degree-aware resampling of the DEGs. No correlation was observed for netPEA that also does not alter pathway properties when constructing the null model. However, the correlation with the fraction of intralinks was much weaker when using BinoX and NEAT with the STRING network, which has other properties. On the other hand, using STRING led to a stronger correlation between FPR and some other network properties, including density of hubs, max degree and median degree.

**Figure 6:**
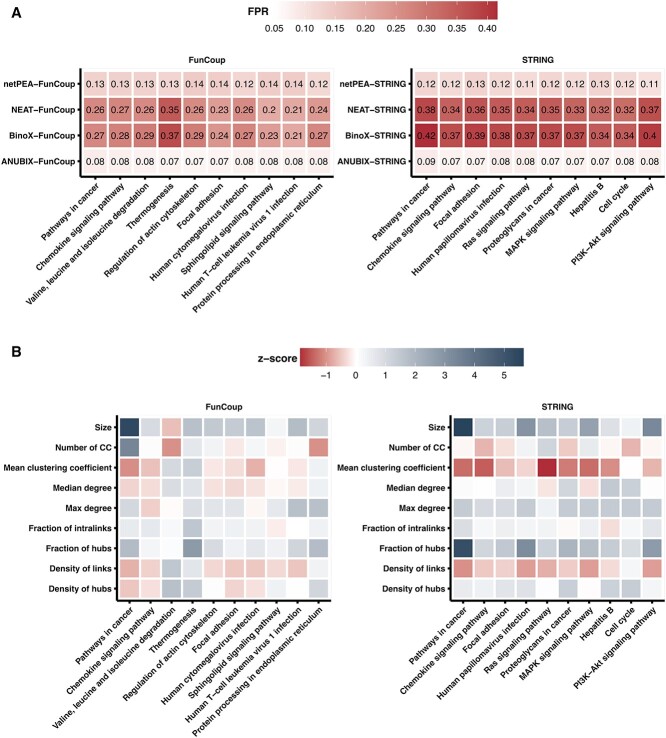
TOP falsely detected pathways in network-based EA methods. The heatmaps show (**A**) FPR and (**B**) the z-score of the network properties of the 10 on average most falsely detected pathways predicted by the NEA methods, both for FunCoup and STRING. The FPR was computed per pathway over 2460 tests under the null hypothesis and is the fraction of *P-*values <0.05. The pathway properties are: size of the pathway; number of connected components (CC); mean average clustering coefficient; median and max pathway degree; fraction of intralinks as the ratio between the number of links within the pathway and the total number of links for the same pathway genes in the network; fraction of hubs as the ratio between the number of hubs in the pathway and the total number of hubs in the network; density of links as the proportion of links within the pathway compared with the total number of possible links; and density of hubs as the proportion of hubs in the pathway compared with the total number of pathway nodes. We defined ‘hubs’ as the 20% highest connected nodes in the network, which was 2587 in FunCoup and 2915 in STRING.

To highlight the pathways most prone to false detection in NEA methods, we isolated the top 10 pathways with the highest average FPR, as depicted in Figure 6A. We then standardized the distributions of network properties to visually represent the extent to which each pathway deviates from the mean (Figure 6B).

### Runtime evaluation

**Figure 7:**
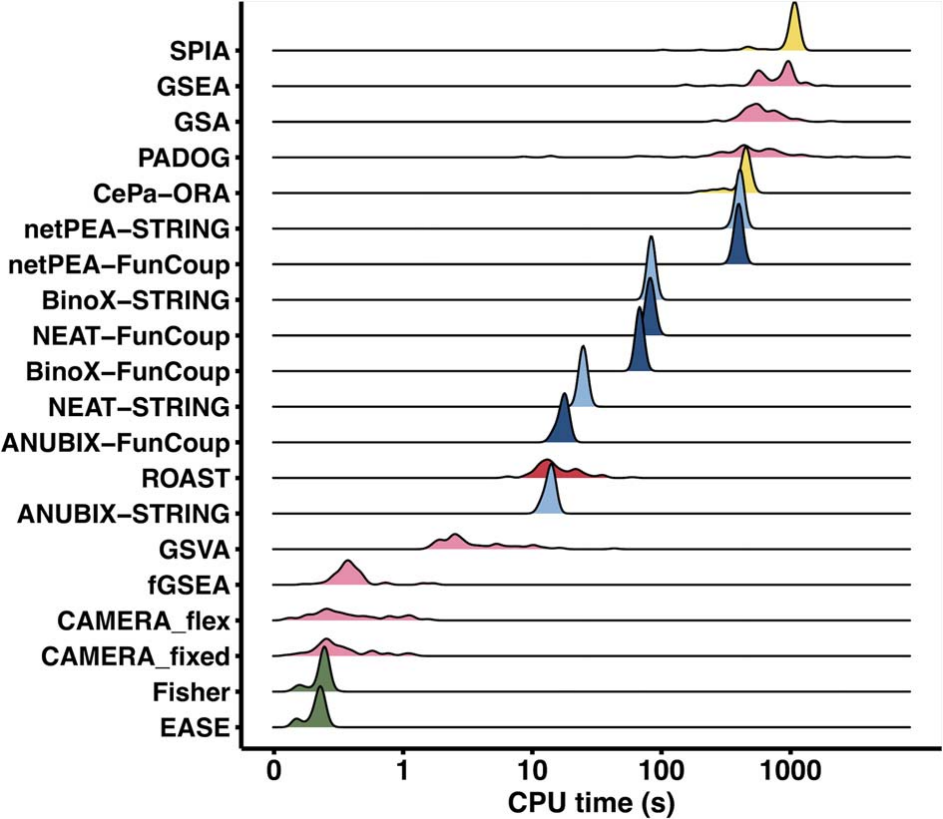
Runtime of enrichment analysis methods. Total CPU times when applying the enrichment methods to the 82 datasets of the benchmark. KEGG pathways were used to define gene sets. The computation was performed on an Apple M1 processor (16GB RAM), with the exception of BinoX that was run on an Intel Core i7–2600 3.40GHz (16GB RAM). GSEA is shown as elapsed time.

We performed scalability tests for each of the benchmarked methods. To detect trends on the runtime over all data in use, we show the runtime per dataset (Figure 7). The analysis was done on the 82 datasets of the benchmark with KEGG as input. GSA, GSEA, ROAST and SPIA have internal parallelization. ANUBIX, BinoX, GSVA, NEAT, netPEA and PADOG allow for parallelization and/or can treat multiple datasets at once. In a battery testing setup, this can vastly reduce the elapsed time. Network pre-processing in ANUBIX and BinoX, as well as KEGG pathways pre-processing in SPIA, was not included in the runtime because this only needs to be done once.

## DISCUSSION AND CONCLUSION

We present a generalized benchmark for evaluating EA methods on transcriptomic data that overcomes several limitations of previous benchmarks. This is the first benchmark that includes all four categories of EA methods, and it also covers a wider range of diseases than previously. While previous EA benchmarks have a strong bias toward cancer, we ensured that no more than half of our datasets were related to cancer. Our benchmark constitutes a further development of the performance assessment of EA methods by introducing the Disease Pathway Network based on connecting KEGG pathways, which overcomes the ‘single target pathway’ shortcoming in a generalized way.

In contrast to previous benchmarks that separately evaluate sensitivity and specificity in pathway EA methods [9, 12, 13], our study uses an overall metric that combines both measures, providing a balanced evaluation of performance. This unified metric allows us to identify the best-performing method that excels in detecting enriched pathways while accurately avoiding non-enriched pathways. However, it is important to recognize the inherent difficulty in estimating the relative challenge of the False Positive (FP) benchmark compared with the True Positive (TP) benchmark. It appears that achieving a higher performance in the false positive (FP) benchmark is easier, but accurately quantifying the exact level of difficulty remains challenging. It is essential for future benchmarking efforts to carefully consider and communicate the trade-offs and challenges associated with both TP and FP benchmarks, providing a comprehensive understanding of method performance across different evaluation metrics.

Another drawback of previous benchmarks is that they were either aimed at methods that take gene sets as input [12, 29] or at methods that use rankings of all genes. By catering for both types of methods, we were able to evaluate a total of 14 EA methods representing all four categories of currently existing approaches: (i) OVA, PGA, PTA and NEA. The selected methods have a history of evaluation in prior benchmark studies [9, 12, 13, 29]. All PGA methods tested in this benchmark are also implemented in EnrichmentBrowser [40]. To our knowledge, this is the first time that all four method categories have been evaluated using a standardized benchmark, providing valuable insights into the strengths and weaknesses of each category.

The benchmark showed that NEA methods have superior sensitivity compared with overlap-based methods, which may suffer from the limited coverage of knowledge-based databases such as KEGG, GO and Reactome [41]. Having a relatively high computational speed, NEA methods are well-suited for analyzing large-scale datasets efficiently. Among the NEA methods, BinoX and NEAT demonstrated the highest sensitivity at the price of high FPRs, which appears to be related to bimodal *P*-value distributions. For BinoX and NEAT, this was partially a result of our approach to treat highly depleted pathways as non-significant enrichments. In addition to that, for pathways yielding a high variance of ESs, statistical tests such as the binomial test (BinoX), the hypergeometric test (NEAT) and the Z-test (netPEA) are prone to underestimate the variance of the null distribution, resulting in a high FPR. ANUBIX, which employs the beta-binomial distribution to model overdispersed crosstalk distributions (see Supplementary Methods, see Supplementary Data available online at http://bib.oxfordjournals.org/), does not suffer from this and showed almost no skewness, achieving a good balance between sensitivity and specificity. Both netPEA and ANUBIX preserve pathway properties in their null model construction, resulting in absence of correlation between FPR and pathway fraction of intralinks in FunCoup. However, to account for biases in the underlying network, it is recommended to consider other factors than the fraction of intralinks, such as hub density and pathway size. In such a way, researchers can heed high FPRs and enhance the reliability of their pathway EA. For users without programming experience, we can suggest user-friendly, web-based applications such as EviNet [42], PathwAX [43] and PathBIX [44] for a streamlined pathway NEA.

Recommendations for pathway analysis users can be tailored based on their specific research objectives. It is essential to highlight that OVA is an extremely conservative approach. The Fisher test, or its modified version, EASE, which is implemented in DAVID [7], although widely used for EA, has shown poor performance in the context of pathway analysis [30]. This can be attributed to the limited gene coverage of pathway databases and the assumption of gene independence, which does not hold true for genes interacting within pathways. As a result, users should exercise caution when relying on platforms using the Fisher test, such as Ingenuity Pathway Analysis [45] or DAVID, as they may produce false negatives due to the aforementioned limitations. Within PGA methods, we could support the ability of PADOG to be best at ranking the target pathways. CAMERA, despite its popularity, performed poorly in our benchmark, with worst results in the flexible configuration, whereas fGSEA/GSEA showed the best G-mean of sensitivity and specificity, with fGSEA being much faster. It can be conveniently executed using clusterProfiler [46], a comprehensive R package for gene set EA. PGA methods are valuable for predicting the direction and impact of biological function alterations. They uniquely consider gene expression changes, offering insights into pathway dynamics. For simulating the effects of expression changes, PTA methods such as SPIA are the best choice. However, a drawback is that pathway topology is not always available, limiting their applicability. On the other hand, if researchers need to analyze arbitrarily defined sets of genes rather than predefined pathways, NEA methods may be more suitable provided that a functional association network is available for the species of interest. NEA methods differ from other approaches by considering the connectivity of DEGs with functional gene sets in a network context. Instead of assuming gene independence, NEA methods leverage genome-wide networks, such as FunCoup [33] and STRING [34], to capture the intricate interactions among genes within pathways. This network-focused analysis allows NEA methods to overcome the limitation of low database coverage leading to substantially higher sensitivity and an increased chance for obtaining biological insights.

In light of the importance of reproducibility in pathway EA, we conducted an assessment of the consistency in enrichment results across independent datasets for the same disease, as illustrated in Supplementary Figure 8 (see Supplementary Data available online at http://bib.oxfordjournals.org/). Our investigation aligns with previous studies emphasizing the significance of reproducibility in this context [47, 48]. Overall, methods exhibiting superior benchmark performance also demonstrate enhanced reproducibility compared with poorly performing methods. In absolute terms, the median overlap between datasets varied greatly between diseases and was found to range between 0 and 0.68 for the Jaccard index and between 0 and 0.91 for the Szymkiewicz−Simpson coefficient.

In summary, choosing the appropriate pathway analysis method depends on the research objectives and the nature of the gene sets being analyzed. Researchers should carefully consider the benefits and limitations of EA methods to make informed decisions for their specific study designs. The limitations highlighted above collectively underscore the need for ongoing benchmarking efforts in the field of pathway EA. Ideally, a web-based platform would be developed to facilitate benchmarking of novel EA methods in a standardized and generalized way.

**Key Points**

- Comprehensive Benchmark. The study presents a generalized benchmark for evaluating enrichment analysis (EA) methods, covering all four categories of current approaches and utilizing a diverse set of 82 gene expression datasets across 26 diseases, with a balanced representation of non-cancer conditions.
- Disease Pathway Network. To improve sensitivity and address single-target pathway limitations, this study introduces the Disease Pathway Network, which links related KEGG pathways for evaluation, enhancing the overall assessment of EA methods.
- Novel Evaluation Approach. A novel approach combining sensitivity and specificity is introduced to evaluate pathway EA, identifying Network Enrichment Analysis methods as top performers over overlap-based methods. Additionally, the study explores the bias in *P*-values generated by different methods using randomized gene expression datasets.

## Supplementary Material

REVISION_davidebuzzao-EAbenchmark_supplementary_bbae069

## Data Availability

We used R (r-project.org) v4.1.0 in a version-controlled conda environment for statistical tests and data visualization. The gene expression datasets can be retrieved from the Gemma database (https://gemma.msl.ubc.ca/). The code to reproduce the results of this benchmark can be found in the repository https://bitbucket.org/sonnhammergroup/eabenchmark.

## Author Biographies

**Davide Buzzao** is a PhD student in bioinformatics at the Department of Biochemistry and Biophysics, Stockholm University (Science For Life Laboratory, SE). His research focuses on the development and application of novel algorithms to disease module detection, pathway analysis and protein network construction.

**Miguel Castresana-Aguirre** is a post-doctoral fellow in cancer genomics at the Department of Oncology-Pathology, Karolinska Institute (SE). His research focuses on the development and application of novel algorithms to understand cell cycle impact in breast cancer from a pan-cancer perspective.

**Dimitri Guala** is a senior scientist in bioinformatics at the Department of Biochemistry and Biophysics, Stockholm University (Science For Life Laboratory, SE). His research focuses on the development and application of novel algorithms for drug repurposing, pathway analysis and protein network construction in the field of network medicine.

**Erik Sonnhammer** is a professor of bioinformatics at the Department of Biochemistry and Biophysics, Stockholm University (Science For Life Laboratory, SE). His research focuses on developing new methods for predicting protein function, including protein network inference and pathway analysis.

## References

[1] Gene Ontology Consortium . Gene ontology consortium: going forward. Nucleic Acids Res 2015;43:D1049–56. 25428369 10.1093/nar/gku1179 PMC4383973

[2] Kanehisa M , Goto S, Sato Y, et al. Data, information, knowledge and principle: back to metabolism in KEGG. Nucleic Acids Res 2014;42:D199–205. 24214961 10.1093/nar/gkt1076 PMC3965122

[3] Croft D , O’Kelly G, Wu G, et al. Reactome: a database of reactions, pathways and biological processes. Nucleic Acids Res 2011;39:D691–7. 21067998 10.1093/nar/gkq1018 PMC3013646

[4] Piñero J , Ramírez-Anguita JM, Saüch-Pitarch J, et al. The DisGeNET knowledge platform for disease genomics: 2019 update. Nucleic Acids Res 2020;48:D845–55. 31680165 10.1093/nar/gkz1021 PMC7145631

[5] Liberzon A , Subramanian A, Pinchback R, et al. Molecular signatures database (MSigDB) 3.0. Bioinformatics 2011;27:1739–40. 21546393 10.1093/bioinformatics/btr260 PMC3106198

[6] Khatri P , Sirota M, Butte AJ. Ten years of pathway analysis: current approaches and outstanding challenges. PLoS Comput Biol 2012;8:e1002375. 22383865 10.1371/journal.pcbi.1002375 PMC3285573

[7] Huang DW , Sherman BT, Lempicki RA. Systematic and integrative analysis of large gene lists using DAVID bioinformatics resources. Nat Protoc 2009;4:44–57. 19131956 10.1038/nprot.2008.211

[8] Subramanian A , Tamayo P, Mootha VK, et al. Gene set enrichment analysis: a knowledge-based approach for interpreting genome-wide expression profiles. Proc Natl Acad Sci U S A 2005;102:15545–50. 16199517 10.1073/pnas.0506580102 PMC1239896

[9] Tarca AL , Bhatti G, Romero R. A comparison of gene set analysis methods in terms of sensitivity, prioritization and specificity. PLoS One 2013;8:e79217. 24260172 10.1371/journal.pone.0079217 PMC3829842

[10] Bayerlová M , Jung K, Kramer F, et al. Comparative study on gene set and pathway topology-based enrichment methods. BMC Bioinformatics 2015;16:334. 26489510 10.1186/s12859-015-0751-5 PMC4618947

[11] Dong X , Hao Y, Wang X, Tian W. LEGO: a novel method for gene set over-representation analysis by incorporating network-based gene weights. Sci Rep 2016;6:18871. 26750448 10.1038/srep18871 PMC4707541

[12] Nguyen T-M , Shafi A, Nguyen T, Draghici S. Identifying significantly impacted pathways: a comprehensive review and assessment. Genome Biol 2019;20:203. 31597578 10.1186/s13059-019-1790-4 PMC6784345

[13] Geistlinger L , Csaba G, Santarelli M, et al. Toward a gold standard for benchmarking gene set enrichment analysis. Brief Bioinform 2021;22:545–56. 32026945 10.1093/bib/bbz158 PMC7820859

[14] Rappaport N , Twik M, Nativ N, et al. MalaCards: a comprehensive automatically-mined database of human diseases. Curr Protoc Bioinformatics 2014;47(1):1.24.1–19. 10.1002/0471250953.bi0124s47 25199789

[15] Lim N , Tesar S, Belmadani M, et al. Curation of over 10 000 transcriptomic studies to enable data reuse. Database 2021;2021:baab006. 33599246 10.1093/database/baab006 PMC7904053

[16] Barrett T , Wilhite SE, Ledoux P, et al. NCBI GEO: archive for functional genomics data sets--update. Nucleic Acids Res 2013;41:D991–5. 23193258 10.1093/nar/gks1193 PMC3531084

[17] Kim CY , Baek S, Cha J, et al. HumanNet v3: an improved database of human gene networks for disease research. Nucleic Acids Res 2022;50:D632–9. 34747468 10.1093/nar/gkab1048 PMC8728227

[18] Fisher, RA. “Statistical methods for research workers.” Breakthroughs in statistics: Methodology and distribution. New York, NY: Springer New York, 1970. 66–70.

[19] Wang JZ , Du Z, Payattakool R, et al. A new method to measure the semantic similarity of GO terms. Bioinformatics 2007;23:1274–81. 17344234 10.1093/bioinformatics/btm087

[20] Hosack DA , Dennis G, Jr, Sherman BT, et al. Identifying biological themes within lists of genes with EASE. Genome Biol 2003;4:R70. 14519205 10.1186/gb-2003-4-10-r70 PMC328459

[21] Wu D , Smyth GK. Camera: a competitive gene set test accounting for inter-gene correlation. Nucleic Acids Res 2012;40:e133. 22638577 10.1093/nar/gks461 PMC3458527

[22] Korotkevich G , Sukhov V, Budin N, et al. Fast gene set enrichment analysis. bioRxiv. 2016;060012.

[23] Efron B , Tibshirani R. On testing the significance of sets of genes. Ann Appl Stat 2007;1:107–29.

[24] Hänzelmann S , Castelo R, Guinney J. GSVA: gene set variation analysis for microarray and RNA-seq data. BMC Bioinformatics 2013;14:7. 23323831 10.1186/1471-2105-14-7 PMC3618321

[25] Tarca AL , Draghici S, Bhatti G, Romero R. Down-weighting overlapping genes improves gene set analysis. BMC Bioinformatics 2012;13:136. 22713124 10.1186/1471-2105-13-136 PMC3443069

[26] Wu D , Lim E, Vaillant F, et al. ROAST: rotation gene set tests for complex microarray experiments. Bioinformatics 2010;26:2176–82. 20610611 10.1093/bioinformatics/btq401 PMC2922896

[27] Gu Z , Wang J. CePa: an R package for finding significant pathways weighted by multiple network centralities. Bioinformatics 2013;29:658–60. 23314125 10.1093/bioinformatics/btt008

[28] Tarca AL , Draghici S, Khatri P, et al. A novel signaling pathway impact analysis. Bioinformatics 2009;25:75–82. 18990722 10.1093/bioinformatics/btn577 PMC2732297

[29] Castresana-Aguirre M , Sonnhammer ELL. Pathway-specific model estimation for improved pathway annotation by network crosstalk. Sci Rep 2020;10:13585. 32788619 10.1038/s41598-020-70239-z PMC7423893

[30] Ogris C , Guala D, Helleday T, Sonnhammer ELL. A novel method for crosstalk analysis of biological networks: improving accuracy of pathway annotation. Nucleic Acids Res 2017;45:e8. 27664219 10.1093/nar/gkw849 PMC5314790

[31] Signorelli M , Vinciotti V, Wit EC. NEAT: an efficient network enrichment analysis test. BMC Bioinformatics 2016;17:352. 27597310 10.1186/s12859-016-1203-6 PMC5011912

[32] Liu L , Ruan J. Network-based pathway enrichment analysis. IEEE International Conference on Bioinformatics and Biomedicine (BIBM), Shanghai, China, 2013, 218–21. 10.1109/BIBM.2013.6732493 PMC4197800 25327472

[33] Persson E , Castresana-Aguirre M, Buzzao D, et al. FunCoup 5: functional association networks in all domains of life, supporting directed links and tissue-specificity. J Mol Biol 2021;433:166835. 33539890 10.1016/j.jmb.2021.166835

[34] Szklarczyk D , Gable AL, Lyon D, et al. STRING v11: protein-protein association networks with increased coverage, supporting functional discovery in genome-wide experimental datasets. Nucleic Acids Res 2019;47:D607–13. 30476243 10.1093/nar/gky1131 PMC6323986

[35] Zhao Z , Xia J, Tastan O, et al. Virus interactions with human signal transduction pathways. Int J Comput Biol Drug Des 2011;4:83–105. 21330695 10.1504/IJCBDD.2011.038658 PMC3407688

[36] Knip M , Siljander H. Autoimmune mechanisms in type 1 diabetes. Autoimmun Rev 2008;7:550–7. 18625444 10.1016/j.autrev.2008.04.008

[37] Notkins AL , Lernmark A. Autoimmune type 1 diabetes: resolved and unresolved issues. J Clin Invest 2001;108:1247–52. 11696564 10.1172/JCI14257 PMC209446

[38] Perz JF , Armstrong GL, Farrington LA, et al. The contributions of hepatitis B virus and hepatitis C virus infections to cirrhosis and primary liver cancer worldwide. J Hepatol 2006;45:529–38. 16879891 10.1016/j.jhep.2006.05.013

[39] Levrero M . Viral hepatitis and liver cancer: the case of hepatitis C. Oncogene 2006;25:3834–47. 16799625 10.1038/sj.onc.1209562

[40] Geistlinger L , Csaba G, Zimmer R. Bioconductor’s EnrichmentBrowser: seamless navigation through combined results of set- & network-based enrichment analysis. BMC Bioinformatics 2016;17:45. 26791995 10.1186/s12859-016-0884-1 PMC4721010

[41] Gable AL , Szklarczyk D, Lyon D, et al. Systematic assessment of pathway databases, based on a diverse collection of user-submitted experiments. Brief Bioinform 2022;23(5):bbac355. 10.1093/bib/bbac355 PMC9487593 36088548

[42] Jeggari A , Alekseenko Z, Petrov I, et al. EviNet: a web platform for network enrichment analysis with flexible definition of gene sets. Nucleic Acids Res 2018;46:W163–70. 29893885 10.1093/nar/gky485 PMC6030852

[43] Ogris C , Castresana-Aguirre M, Sonnhammer ELL. PathwAX II: network-based pathway analysis with interactive visualization of network crosstalk. Bioinformatics 2022;38:2659–60. 35266519 10.1093/bioinformatics/btac153 PMC9048662

[44] Castresana-Aguirre M , Persson E, Sonnhammer ELL. PathBIX-a web server for network-based pathway annotation with adaptive null models. Bioinform Adv 2021;1:vbab010. 36700096 10.1093/bioadv/vbab010 PMC9710673

[45] Krämer A , Green J, Pollard J, Jr, Tugendreich S. Causal analysis approaches in ingenuity pathway analysis. Bioinformatics 2014;30:523–30. 24336805 10.1093/bioinformatics/btt703 PMC3928520

[46] Wu T , Hu E, Xu S, et al. clusterProfiler 4.0: a universal enrichment tool for interpreting omics data. Innovation (Camb) 2021;2:100141. 34557778 10.1016/j.xinn.2021.100141 PMC8454663

[47] Yang Q , Wang S, Dai E, et al. Pathway enrichment analysis approach based on topological structure and updated annotation of pathway. Brief Bioinform 2019;20:168–77. 28968630 10.1093/bib/bbx091

[48] Liu H , Yuan M, Mitra R, et al. CTpathway: a CrossTalk-based pathway enrichment analysis method for cancer research. Genome Med 2022;14:118. 36229842 10.1186/s13073-022-01119-6 PMC9563764

