# Supplementary material for: Benchmarking enrichment analysis methods with the disease pathway network
Source: Brief Bioinform. 2024 Mar 2;25(2):bbae069. doi: 10.1093/bib/bbae069 (PMC10939300; doi:10.1093/bib/bbae069)
Supplement: REVISION_davidebuzzao-EAbenchmark_supplementary_bbae069 [file revision_davidebuzzao-eabenchmark_supplementary_bbae069.pdf]

Supplementary Materials

MATERIALS AND METHODS

GO semantic similarity

For any two pathways that passed the overlap and connectivity tests, the semantic similarity of Gene Ontology (GO) keywords was used as a proxy for their functional similarity. In particular, to compute similarity scores we used the Wang et al. graph-based method [1], which uses the topology of the GO Directed Acyclic Graph (DAG). More specifically we used the function clusterSim from the R package GOSemSim (v2.20.0), with the default semantic contribution factor w_e that was either set to 0.8 or 0.6 depending on the relation. The former value is for “is-a” and the latter for “part-of” relations. The similarity between the GO term A and B is given by the formula,

$$sim_{Wang}(A,B) = \frac{\sum_{t \in T_A \cap T_B} S_A(t) + S_B(t)}{SV(A) + SV(B)} \quad \text{(Eq. 1)}$$

Where the S-value $S_A(t)$ (or $S_B(t)$) scores the closeness of the DAG describing the relations of A (or B) and all its ancestors in GO, with the DAG of a common GO term t between A and B. The S-value of GO term t related to term A is:

$$\begin{cases} S_A(A) = 1 \; if \; t = A \\ S_A(t) = max\{w_e \times S_A(t') | t' \in children \; of \; (t)\} \; if \; t \neq A \end{cases} \quad \text{(Eq. 2)}$$

sim_{Wang} is thus the average of maximum semantic similarities, normalized by the semantic value of GO term A and B, each computed as follows:

$$SV(A) = \sum_{t \in T_A} S_A(t) \quad \text{(Eq. 3)}$$

Because a protein can be annotated by multiple GO terms, the output of the analysis may be a matrix of scores which stores the similarity between the combinations of GO terms annotated for every couple of genes. A combining strategy known as Best-Match Average (BMA) was thus applied to average all best similarities between any combination of genes from two pathways. The analysis was run over all three GO ontologies (i.e. Biological Process, Molecular Function and Cellular Component) excluding Inferred Electronic Annotations (IEA), as IEA are not manually reviewed by experts. An average of the three scores was used as a proxy for the relatedness of the target pathway to any other pathway under study. In such a way we could build a weighted Disease Pathway Network of all target pathways and assess the sensitivity of the EA methods at different levels of confidence, *i.e.* extracting the top 20 connected pathways.

Enrichment Analysis methods

The methods under investigation belonged to four different categories: (i) OVerlap Analysis (OVA), Per-Gene score Analysis (PGA), Pathway Topology Analysis (PTA) and Network Enrichment Analysis (NEA) methods. We report here algorithmic details of each EA category and method in the benchmark. Based on the underlying null hypothesis, an EA method can also be classified as competitive or self-contained [2]. A self-contained method tests if genes in the set of interest are differentially expressed whereas a competitive method tests if they are as differentially expressed as the genes not in the set. For a self-contained test on a gene set, the differential expression of only one of its genes may be enough to reject the null hypothesis that none of the genes in the gene set are different, overall resulting in increased sensitivity. However, self-contained methods might not be helpful in a battery testing setup given the large number of significant reported results. As a consequence, most of the available methods are competitive.

OVerlap Analysis (OVA)

OVerlap Analysis (OVA) methods test the proportion of differentially expressed genes (DEGs) in a functional gene collection against a discrete probability distribution model. One-sided *p*-values are extracted with a Fisher's exact test in Fisher as implemented in [3], and with a modified Fisher's exact test in EASE [4], as implemented in DAVID [5].

Per-Gene score Analysis (PGA)

The most popular approach for analyzing expression data works with each gene separately, employing a statistical model to connect the response to each gene's expression. Each gene undergoes a local statistical test that is used to determine a parametric or permutation-based *p*-value of variation of expression. A global statistical analysis is then implemented to assign an Enrichment Score (ES) to a functional set of genes. For permutation-based methods, the significance of the global statistics for each functional gene set is then evaluated using 2000 sample permutations.

Gene Set Enrichment Analysis (GSEA) uses a Kolmogorov-Smirnov (KS) like running-sum statistics to test if the rankings of the genes in a pathway are significantly biased towards high DE fold-changes or *p*-values [6]. To derive the ES, the running-sum statistic is increased (or decreased) upon encountering a gene inside (or outside) the pathway meanwhile traversing down from the top of the sorted gene list. NES is the maximum deviation from zero (ES) normalized by the size of the gene set. Here fast GSEA (fGSEA) is used to conduct a pre-ranked GSEA [7]. A *p*-value is estimated by permuting the genes in a gene set, which leads to randomly assigned gene sets of the same size.

Gene Set Analysis (GSA) substitutes the KS-like statistic of GSEA with the "maxmean" statistic (i.e. the maximum mean gene scores between the up- or down-regulated component of a gene set), and scales it by its mean and standard deviation to obtain the re-standardized version of the "maxmean" statistic [8]. GSA generates a null distribution for estimating false discovery rates by combining gene and sample permutations.

ROtAtion gene Set Test (ROAST) implements a self-contained test that evaluates the enrichment of a gene set using rotation, a Monte Carlo simulation technology, rather than permutation [9].

Gene Set Variation Analysis (GSVA) computes sample-wise gene set enrichment scores as a function of genes within and outside the gene set, transforming the data from a gene by sample matrix to a gene set by sample matrix [10]. *P*-values are extracted by fitting a generalized linear model (GLM) to each pathway profile of ESs and computing a moderated t-test, by empirical Bayes moderation of the ES standard error towards a global value. As recommended in the manual, we used limma to examine variations in enrichment scores between sample groups.

Correlation Adjusted MEan RAnk gene set test (CAMERA) implements a test that accounts for gene-to-gene correlations [11]. CAMERA was run in two configurations: (i) CAMERA_fixed using a fixed inter-gene correlation of 0.01; (ii) CAMERA_flex using an inter-gene correlation calculated from the input dataset.

Pathway Analysis with Down-weighting of Overlapping Genes (PADOG) pre-computes a table with gene frequencies in the gene sets database and then extracts a ES for each gene set by assigning the mean of absolute values of moderated gene t-scores, weighted by the gene frequencies [12]. In this way, genes

that are found in fewer gene sets have a great impact in the analysis, whereas the contribution of ubiquitous genes is down-weighted. The ES is tested against the null distribution, generated by sample permutations.

Pathway Topology Analysis (PTA)

Signaling Pathway Impact Analysis (SPIA) tests both the over-representation of DEGs and the perturbation of each pathway [13]. The overlap between DEGs and pathway genes is tested with the hypergeometric test. The perturbation of a pathway is determined by propagating fold-changes in expression across the pathway topology. The pathway topology can be represented with a signed directed graph, where the sign represents activation or inhibition. To generate the SPIA pathway input, we used the function makeSPIAdata that processes KEGG xml files. To represent activation and inhibition, we assigned a weight vector β where 1 denotes activation, -1 denotes inhibition and 0 other associations. To assess the significance of a pathway perturbation, SPIA compares the observed net perturbation against the perturbation computed in a randomized scenario in which as many genes as the DEGs are allowed to occupy any position along the pathway and to have any log fold-change within the range of DEGs values. A final *p*-value is returned as the combination of the two analyses, by default using the Fisher method. SPIA does not return a functional enrichment for roughly 1/3 of total pathways, as metabolic pathways in KEGG do not include any activation and/or repression interaction.
Centrality Pathway Over Representation Analysis (CePa-ORA) uses pathways as networks with nodes representing genes [14]. For each pathway, CePa-ORA measures node weights using various centrality metrics: in-degree, out-degree, betweenness, in-largest reach, out-largest reach, and equal weight condition. The final pathway score is calculated by summing the weights of nodes that were differentially expressed in the DE analysis. Pathway significance is assessed using a null distribution derived from permutation of DE genes. Similarly to Nguyen et al. 2019 [15], we selected the final *p*-value for each pathway as the lowest *p*-value among the six derived from the different centrality measurements, as the authors did not indicate a preferred metric.

Network Enrichment Analysis (NEA)

NETwork-based Pathway Enrichment Analysis (netPEA) executes Random Walk with Restarts (RWR) on a network by using DEGs as seed nodes with a predetermined restart probability, by default of 0.5 [16]. At steady state, the resulting probabilities will contain the likelihood of RWR ending in each network node that may be used to derive a distance between the DEGs and all other nodes in the graph. NetPEA averages the probabilities between all genes in a gene set to obtain a final ES. z-scores are calculated to extract *p*-values by extracting a null distribution with a size-aware resampling procedure of size 2000.
Network Enrichment Analysis Test (NEAT) finds crosstalks between the DEGs and a gene set. The crosstalk is defined as the degree of interconnectivity between two gene sets mapped onto a functional association network. NEAT tests enrichment (or depletion) significance against a hypergeometric discrete null distribution of crosstalks [17]. Naïvely, it calculates the expected number of links between two gene sets based on their degrees and then uses the hypergeometric distribution to assess statistical significance. In this way the method assumes that under no functional enrichment the crosstalk follows a hypergeometric distribution, thus the analysis speeds up.
BINOmial null distribution of X-talk (BinoX) evaluates whether the crosstalk between a set of DEGs and a gene set is significantly different from the expected crosstalk, which is estimated from a null model based on randomisation of the network [18]. By default, BinoX extracts N randomized networks based on the Link Assignment and Second Order Conservation method (LA+S), which preserves the original second order topological properties and the network degree distribution within the random model. Once the parameters of the random model have been determined, they may be used to assess the likelihood of observed crosstalk. Because all network connections are binary, it is assumed that crosstalk between any two gene sets follows a binomial distribution. As for NEAT and BinoX, if the expected crosstalk is less than the observed, the *p*-value for the upper tail represents enrichment, whereas the opposite may be interpreted as depletion and is calculated based on the lower tail of the distribution. As depletion is out of the scope of the benchmark, we take 1 - *p*-value as significance for all gene sets with smaller crosstalk than expected.
Adaptive NUll distriButIon of X-talk (ANUBIX) tests the crosstalk between the DEGs and a gene set [19].

Statistical significance is assessed by testing the crosstalk against a per DE gene set adapted beta-binomial distribution estimated by computing a discrete null distribution of crosstalk computed with a degree-aware resampling procedure of size 2000.

KEGG is chosen to compare methods throughout the analysis. To accommodate each network's gene vocabulary, *UP000005640_9606.idmapping* (https://www.uniprot.org/news/2021/02/10/release) and *9606.protein.aliases.v11.5.txt* (https://string-db.org/cgi/download?sessionId=bDexq6ZYgwXq&species_text=Homo+sapiens) are used to map *Gene ID* and *Gene Name* to *Ensembl* gene and protein identifiers*,* for FunCoup and STRING respectively.

Supplementary Figures

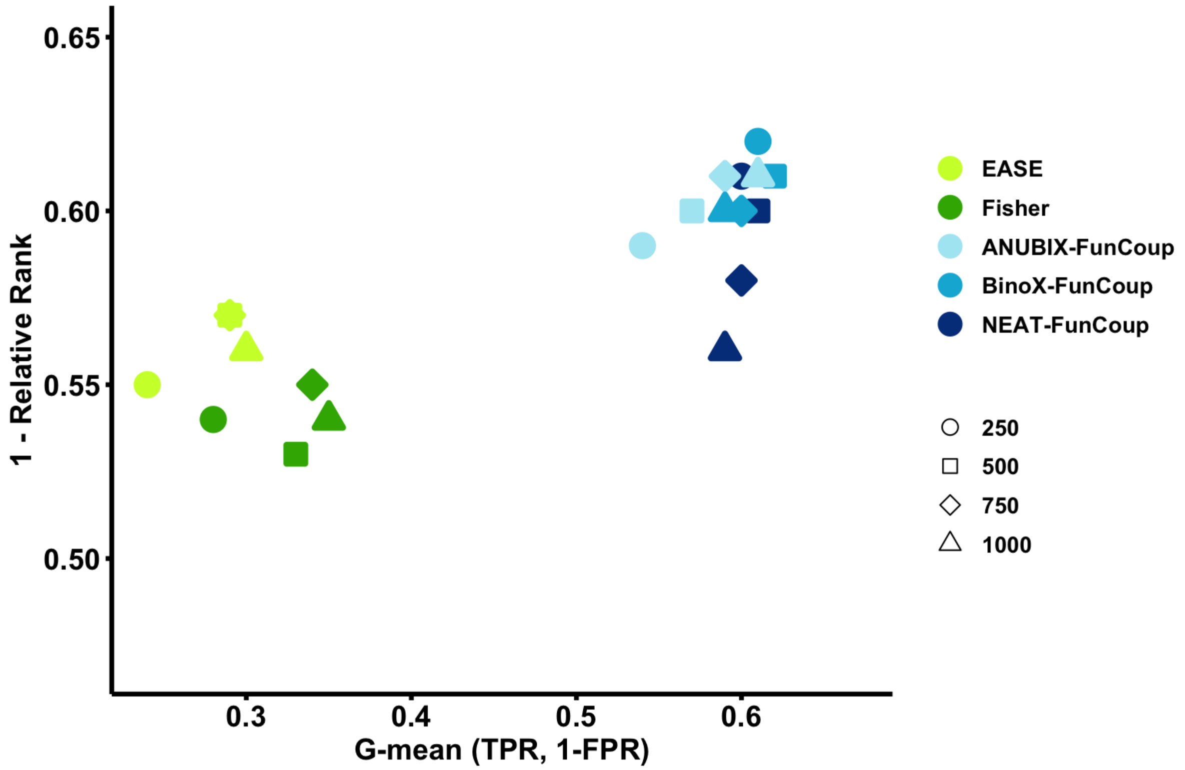

Supplementary Figure 1 - Impact of varying number of DEGs on enrichment results. G-mean of TPR and TNR (1-FPR) is shown versus 1 - relative rank, using the top 20 linked pathways per target pathway in the Disease Pathway Network, at different numbers of DEGs. An ideal method would rank the related pathways for a specific dataset at the top of enrichments and would assign significant *p*-values to only them, which would give a score of (1, 1) and a placement in the top right corner. The group of colors correspond to method categories: greens for OVA, and blues for FunCoup-based NEA methods implementations. The shape corresponds to the size of DEGs in input.

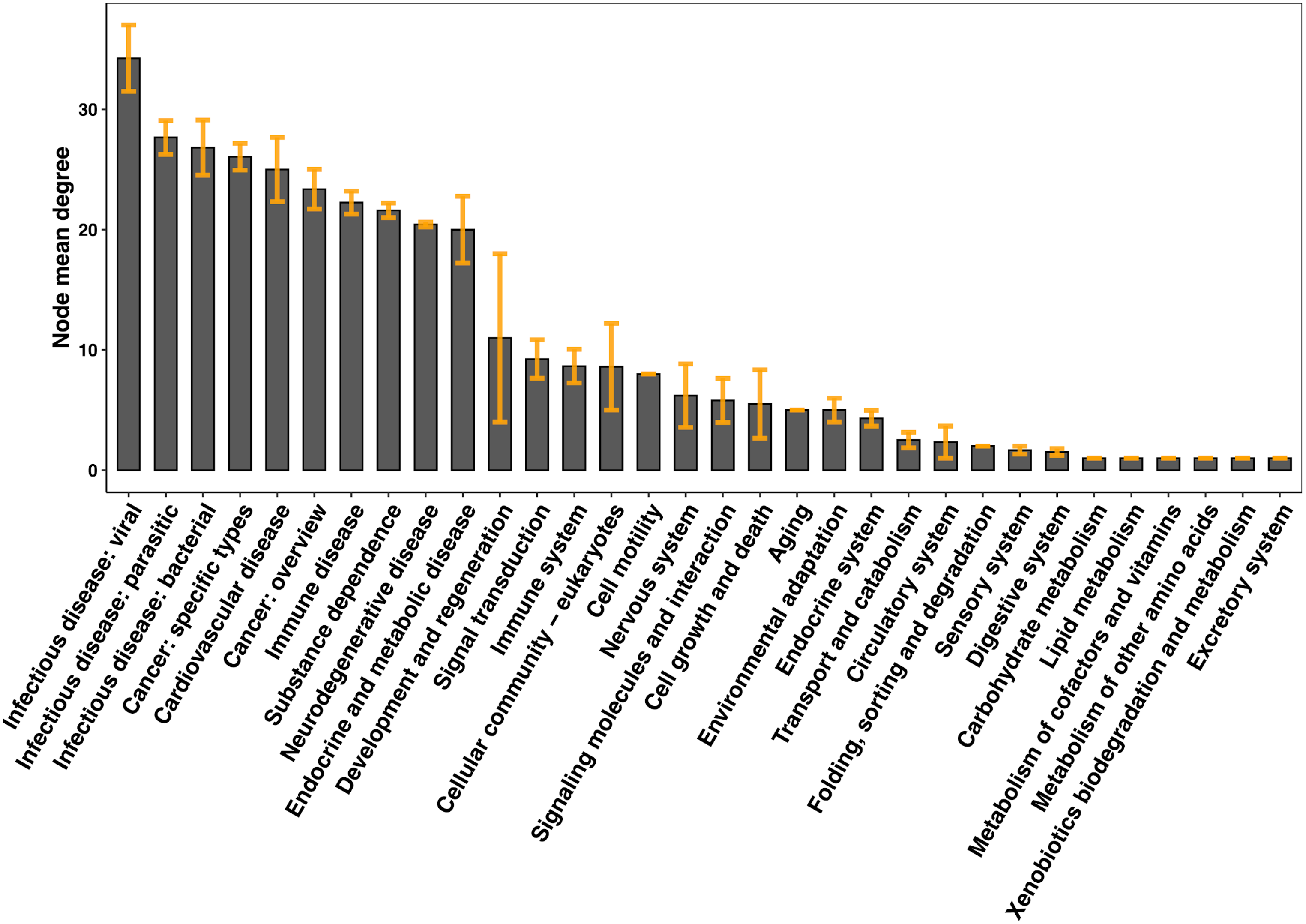

Supplementary Figure 2 - Average node degree distribution of KEGG subclasses of pathways. The height of each bar represents the mean node degree between pathways within the same KEGG subclass in the Disease Pathway Network, using the top 20 linked pathways per target pathway. Error bars indicate the standard error.

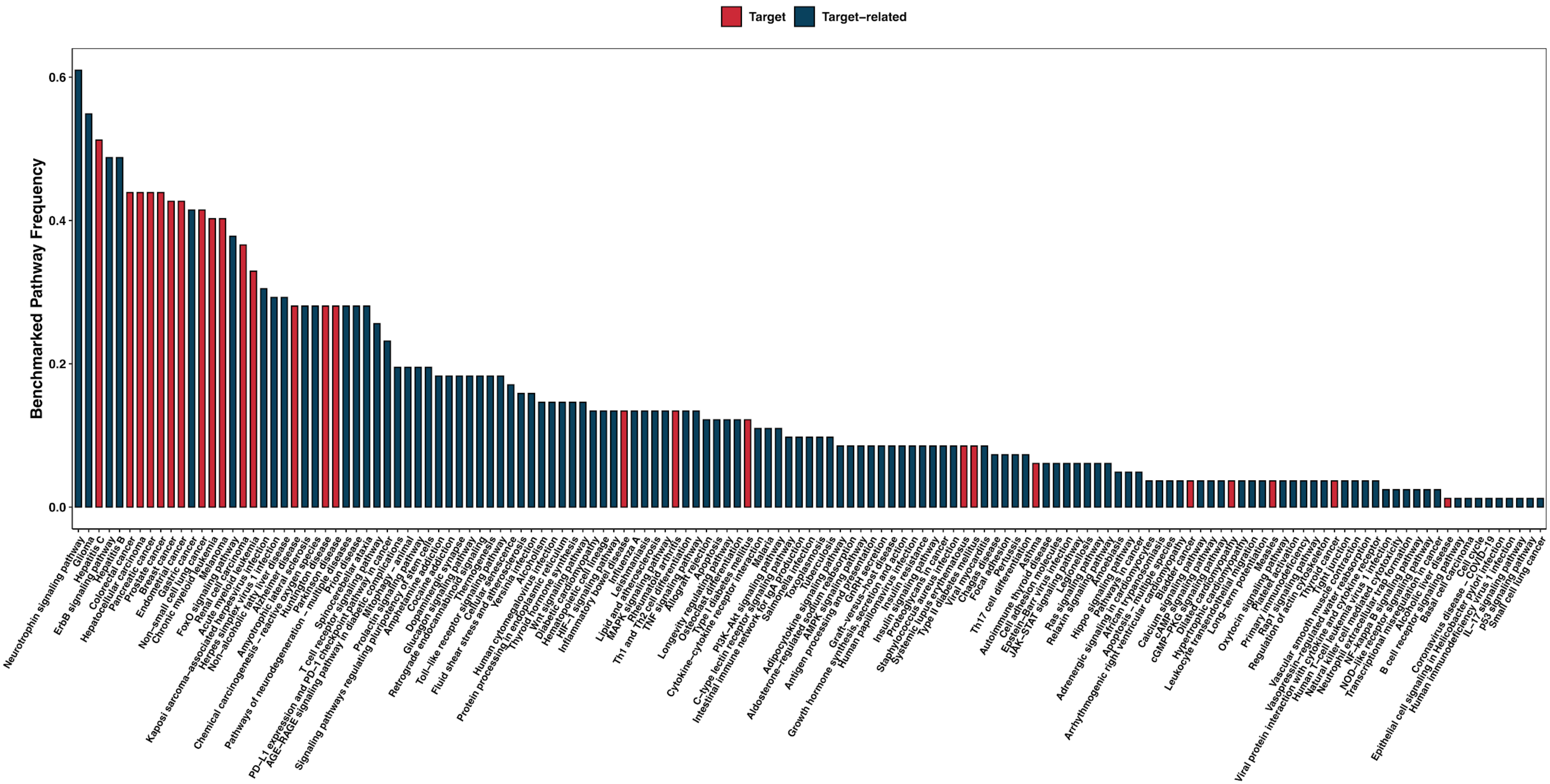

Supplementary Figure 3 - Fraction of times a pathway is tested in the benchmark. The height of each bar represents the proportion of datasets and disease pathway networks where each pathway was evaluated. As we employ disease pathway networks tailored for each disease and have multiple datasets per disease, there are instances where a pathway may be tested more than once. This occurs if the pathway is connected to more than one disease or to one disease for which we have multiple datasets. On the other hand, a pathway may be tested only once if it is unique to a specific disease for which we have only one dataset.

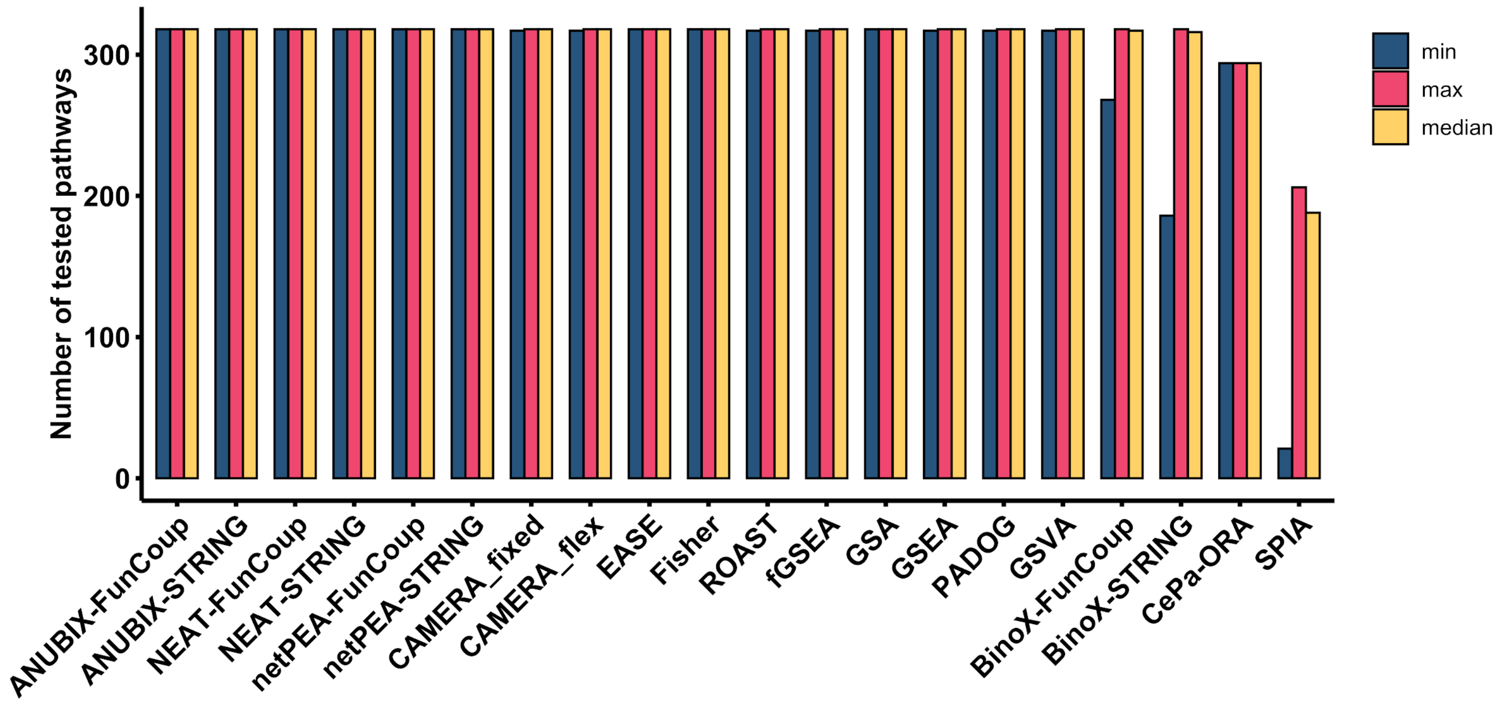

Supplementary Figure 4 - Number of tested pathways in the positive benchmark. This histogram illustrates the variation in the number of tested pathways within the positive benchmark, highlighting the minimum (min), maximum (max), and median average values.

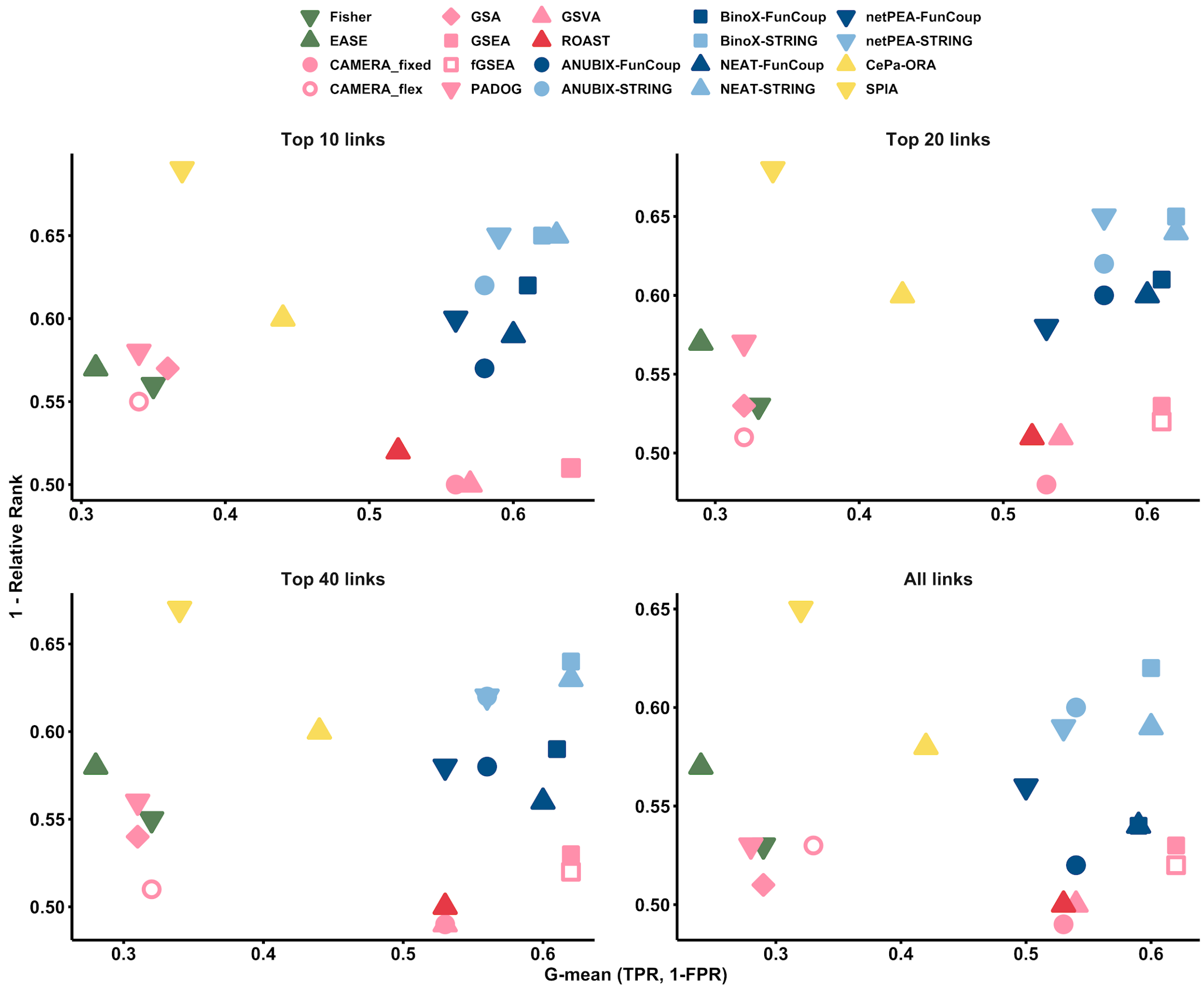

Supplementary Figure 5 - Summary of performances of 14 EA methods at varying number of target-related pathways. G-mean of TPR and TNR (1-FPR) is shown versus 1 - relative rank, using the top 10, 20, 40 and all linked pathways per target pathway in the Disease Pathway Network. An ideal method would rank the related pathways for a specific dataset at the top of enrichments and would assign significant *p*-values to only them, which would give a score of (1, 1) and a placement in the top right corner. In the figure, colors refer to EA categories: green for OVA, pink for PGA with light and dark shades for competitive and self-contained null hypothesis, yellow for PTA, and blue for NEA methods with light and dark shades for STRING-/FunCoup-based implementations.

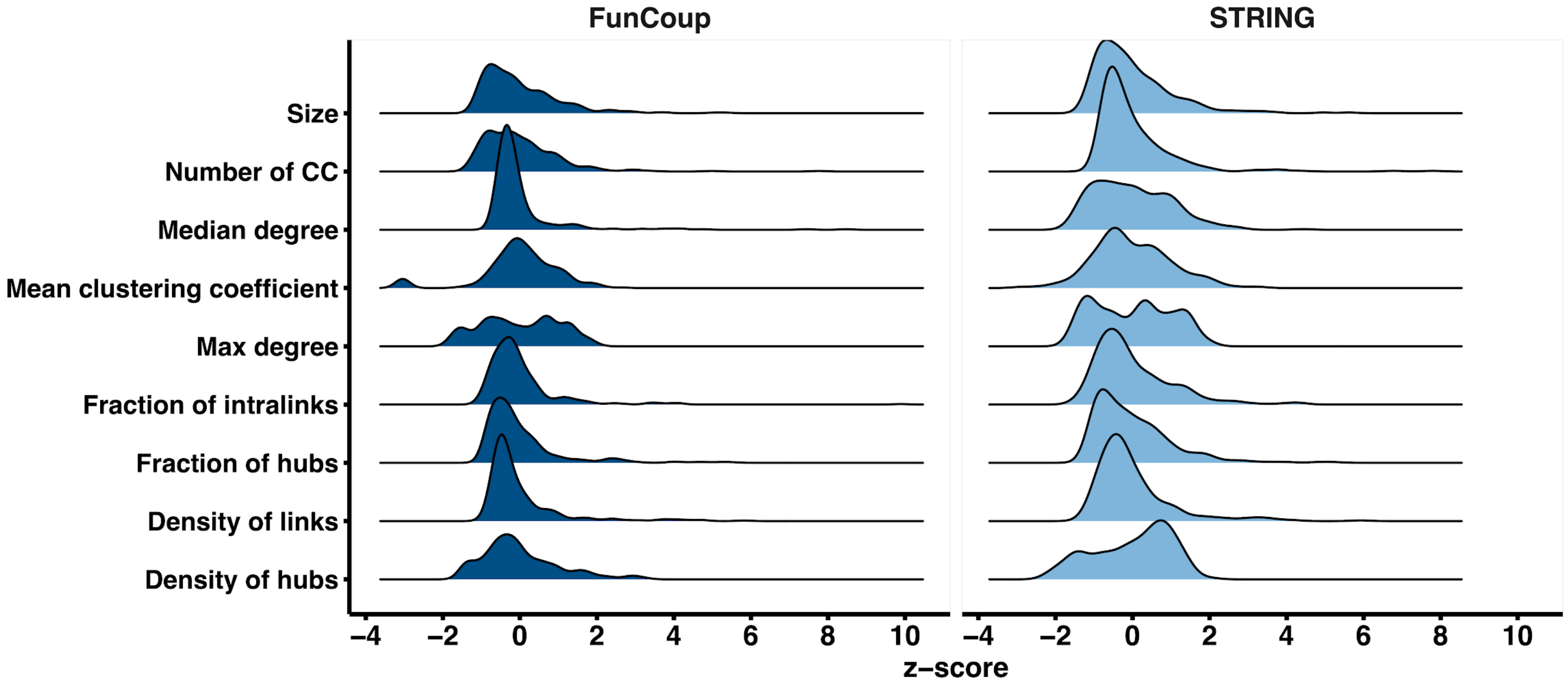

Supplementary Figure 6 - Distribution of standardized network properties of KEGG pathways in FunCoup and STRING. The pathway properties are: size of the pathway; number of connected components (CC); mean average clustering coefficient; median and max pathway degree; fraction of intralinks as the ratio between the number of links within the pathway and the total number of links for the same pathway genes in the network; fraction of hubs as the ratio between the number of hubs in the pathway and the total number of hubs in the network; density of links as the proportion of links within the pathway compared to the total number of possible links; density of hubs as the proportion of hubs in the pathway compared to the total number of pathway nodes. We defined "hubs" as the 20% highest connected nodes in the network, which was 2587 in FunCoup and 2915 in STRING.

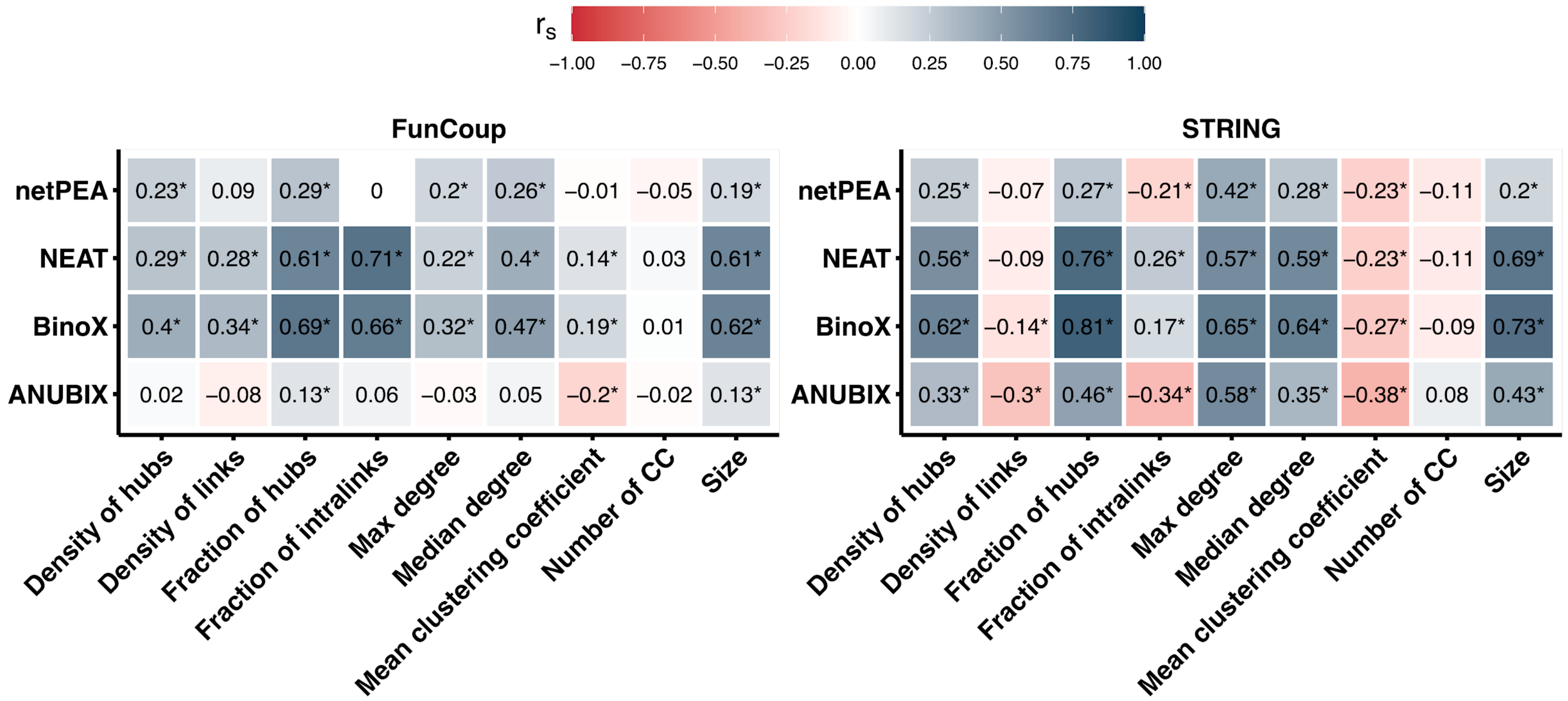

Supplementary Figure 7 - Correlation between pathway network properties and false positive rate. The heatmap shows Spearman correlations for a selection of network properties against the False Positive Rate (FPR) of KEGG pathways as predicted by network-based EA methods with FunCoup and STRING as underlying functional association networks. See description of Supplementary Figure 5 for details about computing the network properties. *P*-values of the correlations were estimated with a test for correlation between paired samples and are reported in Supplementary Table 5. Significant correlations with *p*-values below 0.05 are shown in the figure with a *. The FPR was evaluated on the benchmark datasets by resampling from the genome to generate random gene labels on the 82 datasets. This resampling process was repeated 30 times per dataset, resulting in a total of 2460 resampling tests per pathway. FPR was computed as the complement to 1 of TNR (eq. 7).

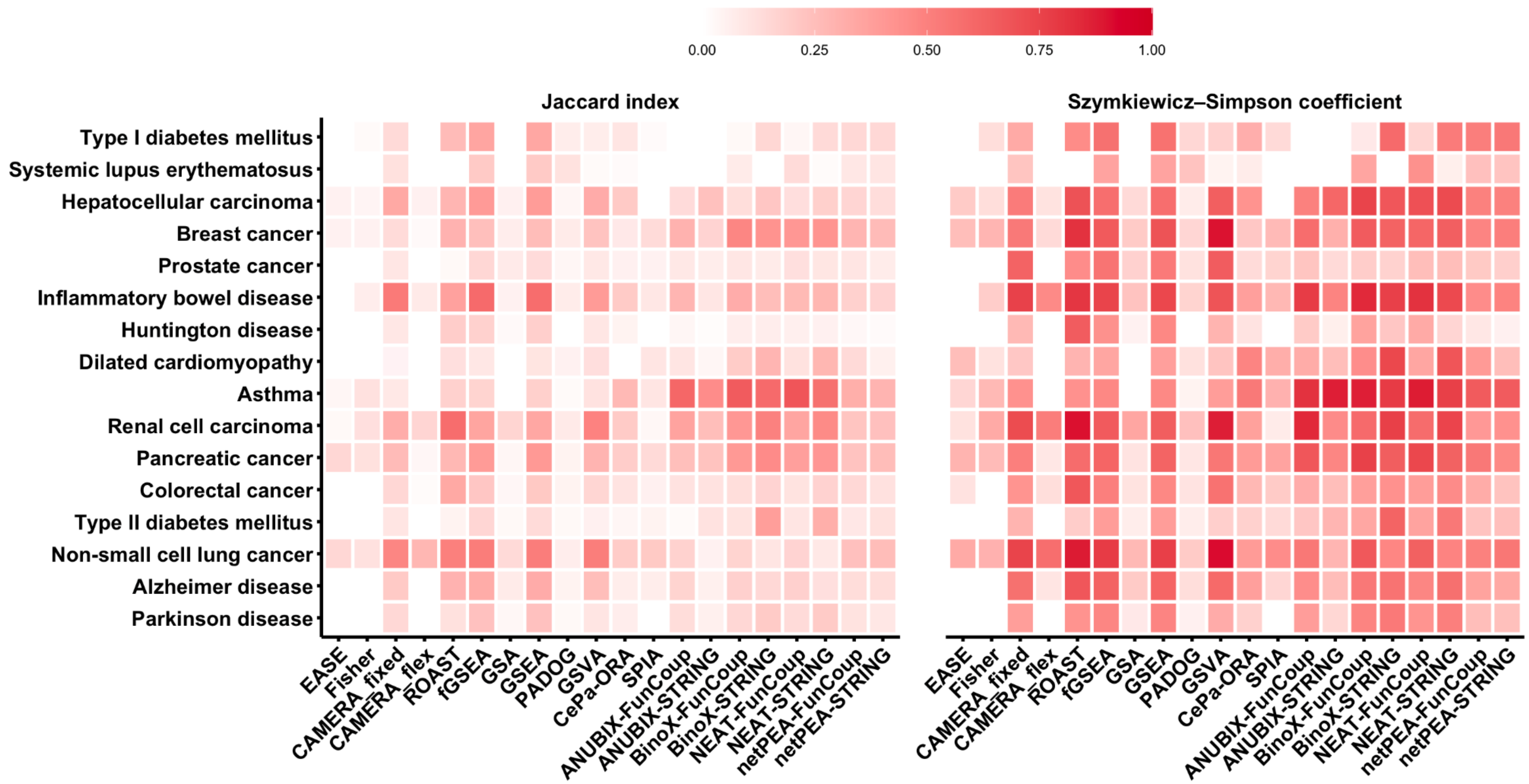

Supplementary Figure 8 - Reproducibility in enrichment results. The heatmap shows median Jaccard index and Szymkiewicz-Simpson coefficients of significantly enriched pathways (p-value < 0.05) between different datasets for the same disease. The Jaccard index and the Szymkiewicz-Simpson coefficients are defined as the number of overlapping enrichments divided by the union or the minimum number of enrichments, respectively. Diseases are sorted by number of datasets in the benchmark, with 12 datasets for “Parkinson disease” and 2 for “Type I diabetes mellitus”.

Supplementary Tables

Supplementary Table 1 - Overview of data. 82 well-characterized DNA-microarray and RNA-Seq datasets in which a pathway is assumed to be prior knowledge under the assumption of an association with the tested condition were retrieved from GEMMA, 76 of which are DNA-microarray and 6 RNA-Seq (*). GEO accession is reported. The number of samples was chosen to be at least 3 per condition. Differentially Expressed Genes (DEGs) are genes with Benjamini-Hochberg (BH) FDR-corrected *p*-values of less than 0.1 and of less than 0.2 (**†**). The number of DEGs was set to be no larger than 500. The number of control and case samples, as well as up- and down-regulated genes, is shown within parentheses under respective columns in the table.

GEO	Disease/Target pathway	Nr. of samples	Genome coverage	Nr. of DEGs	Batch effect
**GSE14858**	Acute myeloid leukemia	39 (20/19)	18337	500 (293/207)	Corrected
**GSE28619**	Alcoholic liver disease	22 (15/7)	18464	500 (331/169)	Not detected
**GSE12685**	Alzheimer disease	13 (6/7)	11141	500 (222/278)	Corrected
**GSE28146**	Alzheimer disease	29 (21/8)	17912	500 (171/329)	Corrected
**GSE36980**	Alzheimer disease	79 (32/47)	17837	500 (141/359)	Corrected
**GSE37263†**	Alzheimer disease	16 (8/8)	15133	500 (169/331)	Corrected
**GSE39420**	Alzheimer disease	21 (14/7)	17947	500 (162/338)	Not detected
**GSE4757**	Alzheimer disease	20 (10/10)	18801	94 (65/29)	Corrected
**GSE95587***	Alzheimer disease	117 (84/33)	18297	500 (211/289)	Not detected
**GSE97760**	Alzheimer disease	19 (9/10)	10779	500 (397/103)	Not detected
**GSE19187**	Asthma	38 (27/11)	17803	500 (366/134)	Corrected
**GSE23552**	Asthma	39 (26/13)	15210	500 (290/210)	Corrected
**GSE27011**	Asthma	54 (36/18)	17634	500 (273/227)	Corrected
**GSE31189**	Bladder cancer	92 (52/40)	18649	120 (50/70)	Corrected
**GSE10810**	Breast cancer	58 (31/27)	18543	500 (77/423)	Corrected
**GSE26304**	Breast cancer	115 (109/6)	16638	500 (107/393)	Corrected
**GSE43754**	Chronic myeloid leukemia	19 (9/10)	15020	500 (308/192)	Not detected

**GSE10715**	Colorectal cancer	30 (19/11)	18210	500 (244/256)	Corrected
**GSE13067**	Colorectal cancer	72 (11/61)	18722	500 (279/221)	Corrected
**GSE31737**	Colorectal cancer	79 (40/39)	15188	500 (292/208)	Corrected
**GSE4107**	Colorectal cancer	22 (12/10)	18618	500 (297/203)	Corrected
**GSE49355**	Colorectal cancer	56 (39/17)	11233	500 (192/308)	Corrected
**GSE50117**	Colorectal cancer	18 (9/9)	10100	500 (311/189)	Not detected
**GSE16499**	Dilated cardiomyopathy	30 (15/15)	15095	500 (218/282)	Corrected
**GSE3586**	Dilated cardiomyopathy	28 (13/15)	3765	500 (231/269)	Not detected
**GSE42955**	Dilated cardiomyopathy	29 (24/5)	17854	500 (169/331)	Corrected
**GSE36389**	Endometrial cancer	19 (13/6)	11210	500 (151/349)	Corrected
**GSE40184**	Hepatitis C	18 (10/8)	11099	500 (240/260)	Corrected
**GSE38476**	Hepatocellular carcinoma	20 (10/10)	13131	500 (288/212)	Not detected
**GSE54236**	Hepatocellular carcinoma	160 (80/80)	16823	500 (300/200)	Corrected
**GSE45516**	Huntington disease	9 (6/3)	18331	500 (339/161)	Not detected
**GSE64810***	Huntington disease	69 (20/49)	16046	500 (338/162)	Not detected
**GSE73655**	Huntington disease	20 (13/7)	19881	65 (39/26)	Not detected
**GSE14580**	Inflammatory bowel disease	14 (8/6)	18612	500 (273/227)	Corrected
**GSE22619**	Inflammatory bowel disease	20 (10/10)	18819	500 (201/299)	Corrected
**GSE36807**	Inflammatory bowel disease	35 (28/7)	18554	500 (193/307)	Not detected
**GSE5808**	Measles	18 (15/3)	11156	500 (135/365)	Corrected
**GSE15605**	Melanoma	74 (58/16)	18045	500 (145/355)	Not detected
**GSE18842**	Non-small cell lung cancer	91 (46/45)	18683	500 (205/295)	Corrected
**GSE19188**	Non-small cell lung cancer	156 (91/65)	18484	500 (141/359)	Corrected
**GSE19804**	Non-small cell lung cancer	119 (59/60)	18682	500 (129/371)	Corrected
**GSE20189**	Non-small cell lung cancer	162 (81/81)	10949	500 (188/312)	Corrected
**GSE21933**	Non-small cell lung cancer	42 (21/21)	18941	500 (161/339)	Not detected

**GSE27262**	Non-small cell lung cancer	50 (25/25)	18369	500 (100/400)	Corrected
**GSE52248***	Non-small cell lung cancer	18 (12/6)	15210	500 (153/347)	Not detected
**GSE16515**	Pancreatic cancer	52 (36/16)	18361	500 (374/126)	Corrected
**GSE18670**	Pancreatic cancer	23 (11/12)	18656	320 (187/133)	Corrected
**GSE23397**	Pancreatic cancer	21 (15/6)	15168	500 (218/282)	Corrected
**GSE28735**	Pancreatic cancer	90 (45/45)	18141	500 (378/122)	Not detected
**GSE42952**	Pancreatic cancer	23 (11/12)	18655	500 (131/369)	Not detected
**GSE18838**	Parkinson disease	28 (17/11)	14894	500 (134/366)	Corrected
**GSE19587†**	Parkinson disease	22 (12/10)	11219	500 (164/336)	Corrected
**GSE20141**	Parkinson disease	18 (10/8)	17802	500 (452/48)	Corrected
**GSE20146†**	Parkinson disease	19 (10/9)	17870	132 (25/107)	Corrected
**GSE20163**	Parkinson disease	17 (8/9)	11335	500 (177/323)	Corrected
**GSE20164†**	Parkinson disease	11 (6/5)	11239	195 (64/131)	Corrected
**GSE20291**	Parkinson disease	35 (15/20)	11341	500 (227/273)	Corrected
**GSE20292**	Parkinson disease	29 (11/18)	11335	500 (232/268)	Corrected
**GSE20314†**	Parkinson disease	8 (4/4)	11118	58 (38/20)	Corrected
**GSE20333†**	Parkinson disease	12 (6/6)	6756	298 (226/72)	Corrected
**GSE7621**	Parkinson disease	25 (16/9)	18430	500 (204/296)	Corrected
**GSE90514***	Parkinson disease	8 (4/4)	14486	128 (84/44)	Not detected
**GSE11682†**	Prostate cancer	33 (17/16)	19547	59 (28/31)	Corrected
**GSE22260***	Prostate cancer	30 (20/10)	15837	500 (329/171)	Not detected
**GSE30521**	Prostate cancer	22 (17/5)	15150	500 (292/208)	Corrected
**GSE10927**	Renal cell carcinoma	64 (54/10)	18392	500 (94/406)	Corrected
**GSE15641**	Renal cell carcinoma	92 (69/23)	11227	500 (103/397)	Corrected
**GSE33371**	Renal cell carcinoma	64 (54/10)	18392	500 (94/406)	Corrected
**GSE53757**	Renal cell carcinoma	143 (71/72)	18268	500 (173/327)	Corrected

**GSE55235**	Rheumatoid arthritis	20 (10/10)	11131	500 (320/180)	Corrected
**GSE30153**	Systemic lupus erythematosus	26 (17/9)	18065	20 (5/15)	Corrected
**GSE50635**	Systemic lupus erythematosus	48 (32/16)	17642	293 (214/79)	Corrected
**GSE48850***	Thyroid cancer	11 (6/5)	15665	500 (249/251)	Not detected
**GSE10586†**	Type I diabetes mellitus	27 (12/15)	18023	25 (18/7)	Corrected
**GSE41762**	Type I diabetes mellitus	77 (20/57)	18094	500 (303/197)	Corrected
**GSE12643†**	Type II diabetes mellitus	20 (10/10)	7513	62 (49/13)	Corrected
**GSE13760†**	Type II diabetes mellitus	21 (10/11)	10994	29 (10/19)	Corrected
**GSE15653**	Type II diabetes mellitus	18 (13/5)	11157	500 (303/197)	Corrected
**GSE20966**	Type II diabetes mellitus	20 (10/10)	18819	240 (111/129)	Corrected
**GSE21340**	Type II diabetes mellitus	20 (5/15)	3499	444 (176/268)	Corrected
**GSE38642**	Type II diabetes mellitus	63 (9/54)	18105	500 (189/311)	Corrected
**GSE40234**	Type II diabetes mellitus	62 (34/28)	19098	452 (202/250)	Corrected
**GSE14858**	Acute myeloid leukemia	39 (20/19)	18337	500 (293/207)	Corrected
**GSE28619**	Alcoholic liver disease	22 (15/7)	18464	500 (331/169)	Not detected
**GSE12685**	Alzheimer disease	13 (6/7)	11141	500 (222/278)	Corrected

Supplementary Table 2 - Size of disease pathway networks at different levels of confidence. We report the details of the disease/target pathways according to KEGG (*i.e.* pathway name, pathway id and pathway subclass), and the size of their network of interactions with all other pathways ("All", including the target pathway itself). As we assess the sensitivity of the methods by using the disease pathway network at different levels of confidence, we report the number of all linked pathways and for different top cutoffs (10,20,40). See "Disease pathway network construction" in the Methods section for details about the network construction process.

Pathway name	Pathway ID	Pathway subclass (Human diseases)	Top pathways			
			10	20	40	All
**Acute myeloid leukemia**	hsa05221	Cancer: specific types	11	21	43	129
**African trypanosomiasis**	hsa05143	Infectious disease: parasitic	11	21	42	73
**AGE-RAGE signaling pathway in diabetic complications**	hsa04933	Endocrine and metabolic disease	12	21	42	143
**Alcoholic liver disease**	hsa04936	Endocrine and metabolic disease	11	21	41	64
**Alcoholism**	hsa05034	Substance dependence	11	21	41	106
**Allograft rejection**	hsa05330	Immune disease	11	21	41	53
**Alzheimer disease**	hsa05010	Neurodegenerative disease	11	21	41	82
**Amoebiasis**	hsa05146	Infectious disease: parasitic	11	21	41	98
**Amphetamine addiction**	hsa05031	Substance dependence	11	21	41	75
**Amyotrophic lateral sclerosis**	hsa05014	Neurodegenerative disease	11	21	24	24
**Arrhythmogenic right ventricular cardiomyopathy**	hsa05412	Cardiovascular disease	11	21	27	27
**Asthma**	hsa05310	Immune disease	11	21	41	41
**Autoimmune thyroid disease**	hsa05320	Immune disease	11	22	41	52
**Bacterial invasion of epithelial cells**	hsa05100	Infectious disease: bacterial	11	21	41	80
**Basal cell carcinoma**	hsa05217	Cancer: specific types	11	21	38	38
**Bladder cancer**	hsa05219	Cancer: specific types	11	21	41	108
**Breast cancer**	hsa05224	Cancer: specific types	11	21	42	109
**Central carbon metabolism in cancer**	hsa05230	Cancer: overview	11	21	41	95
**Chagas disease**	hsa05142	Infectious disease: parasitic	11	22	41	139
**Chemical carcinogenesis - DNA adducts**	hsa05204	Cancer: overview	11	15	15	15

**Chemical carcinogenesis - reactive oxygen species**	hsa05208	Cancer: overview	11	21	43	73
**Chemical carcinogenesis - receptor activation**	hsa05207	Cancer: overview	12	22	41	106
**Choline metabolism in cancer**	hsa05231	Cancer: overview	11	21	41	129
**Chronic myeloid leukemia**	hsa05220	Cancer: specific types	11	22	41	129
**Cocaine addiction**	hsa05030	Substance dependence	11	21	41	82
**Colorectal cancer**	hsa05210	Cancer: specific types	11	21	41	128
**Coronavirus disease - COVID-19**	hsa05171	Infectious disease: viral	11	21	41	98
**Cushing syndrome**	hsa04934	Endocrine and metabolic disease	11	21	41	92
**Diabetic cardiomyopathy**	hsa05415	Cardiovascular disease	12	21	41	50
**Dilated cardiomyopathy**	hsa05414	Cardiovascular disease	11	21	41	66
**Endometrial cancer**	hsa05213	Cancer: specific types	11	21	42	122
**Epithelial cell signaling in Helicobacter pylori infection**	hsa05120	Infectious disease: bacterial	12	22	42	105
**Epstein-Barr virus infection**	hsa05169	Infectious disease: viral	11	21	41	109
**Fluid shear stress and atherosclerosis**	hsa05418	Cardiovascular disease	11	21	41	120
**Gastric cancer**	hsa05226	Cancer: specific types	11	21	41	101
**Glioma**	hsa05214	Cancer: specific types	11	21	41	129
**Graft-versus-host disease**	hsa05332	Immune disease	12	21	41	53
**Hepatitis B**	hsa05161	Infectious disease: viral	11	21	41	124
**Hepatitis C**	hsa05160	Infectious disease: viral	12	21	41	128
**Hepatocellular carcinoma**	hsa05225	Cancer: specific types	11	21	41	110
**Herpes simplex virus 1 infection**	hsa05168	Infectious disease: viral	11	21	41	97
**Human cytomegalovirus infection**	hsa05163	Infectious disease: viral	12	21	41	147
**Human immunodeficiency virus 1 infection**	hsa05170	Infectious disease: viral	11	21	41	120
**Human papillomavirus infection**	hsa05165	Infectious disease: viral	11	23	41	120
**Human T-cell leukemia virus 1 infection**	hsa05166	Infectious disease: viral	11	21	41	114
**Huntington disease**	hsa05016	Neurodegenerative disease	11	21	23	23

**Hypertrophic cardiomyopathy**	hsa05410	Cardiovascular disease	11	21	41	43
**Inflammatory bowel disease**	hsa05321	Immune disease	11	22	41	79
**Influenza A**	hsa05164	Infectious disease: viral	11	21	41	105
**Insulin resistance**	hsa04931	Endocrine and metabolic disease	11	21	41	119
**Kaposi sarcoma-associated herpesvirus infection**	hsa05167	Infectious disease: viral	11	21	41	132
**Legionellosis**	hsa05134	Infectious disease: bacterial	11	21	41	62
**Leishmaniasis**	hsa05140	Infectious disease: parasitic	11	22	41	99
**Lipid and atherosclerosis**	hsa05417	Cardiovascular disease	13	23	42	121
**Malaria**	hsa05144	Infectious disease: parasitic	11	21	41	58
**Maturity onset diabetes of the young**	hsa04950	Endocrine and metabolic disease	3	3	3	3
**Measles**	hsa05162	Infectious disease: viral	11	21	41	113
**Melanoma**	hsa05218	Cancer: specific types	11	21	41	114
**MicroRNAs in cancer**	hsa05206	Cancer: overview	11	22	41	86
**Morphine addiction**	hsa05032	Substance dependence	11	21	41	60
**Nicotine addiction**	hsa05033	Substance dependence	11	21	23	23
**Non-alcoholic fatty liver disease**	hsa04932	Endocrine and metabolic disease	11	21	41	96
**Non-small cell lung cancer**	hsa05223	Cancer: specific types	12	21	41	129
**Pancreatic cancer**	hsa05212	Cancer: specific types	11	21	41	135
**Parkinson disease**	hsa05012	Neurodegenerative disease	11	21	28	28
**Pathogenic Escherichia coli infection**	hsa05130	Infectious disease: bacterial	12	21	42	105
**Pathways in cancer**	hsa05200	Cancer: overview	11	21	41	73
**Pathways of neurodegeneration - multiple diseases**	hsa05022	Neurodegenerative disease	11	21	41	52
**PD-L1 expression and PD-1 checkpoint pathway in cancer**	hsa05235	Cancer: overview	11	21	42	131
**Pertussis**	hsa05133	Infectious disease: bacterial	11	21	42	108
**Primary immunodeficiency**	hsa05340	Immune disease	11	21	26	26
**Prion disease**	hsa05020	Neurodegenerative disease	11	21	41	77

**Prostate cancer**	hsa05215	Cancer: specific types	11	22	42	115
**Proteoglycans in cancer**	hsa05205	Cancer: overview	11	21	41	121
**Renal cell carcinoma**	hsa05211	Cancer: specific types	11	21	41	129
**Rheumatoid arthritis**	hsa05323	Immune disease	11	21	41	67
**Salmonella infection**	hsa05132	Infectious disease: bacterial	11	21	41	114
**Shigellosis**	hsa05131	Infectious disease: bacterial	11	22	41	114
**Small cell lung cancer**	hsa05222	Cancer: specific types	11	21	41	109
**Spinocerebellar ataxia**	hsa05017	Neurodegenerative disease	11	21	41	58
**Staphylococcus aureus infection**	hsa05150	Infectious disease: bacterial	11	21	31	31
**Systemic lupus erythematosus**	hsa05322	Immune disease	11	21	39	39
**Thyroid cancer**	hsa05216	Cancer: specific types	11	21	41	108
**Toxoplasmosis**	hsa05145	Infectious disease: parasitic	11	21	42	113
**Transcriptional misregulation in cancer**	hsa05202	Cancer: overview	11	21	36	36
**Tuberculosis**	hsa05152	Infectious disease: bacterial	11	21	41	108
**Type I diabetes mellitus**	hsa04940	Endocrine and metabolic disease	11	21	41	43
**Type II diabetes mellitus**	hsa04930	Endocrine and metabolic disease	11	21	41	125
**Vibrio cholerae infection**	hsa05110	Infectious disease: bacterial	11	21	42	68
**Viral carcinogenesis**	hsa05203	Cancer: overview	11	21	41	93
**Viral myocarditis**	hsa05416	Cardiovascular disease	12	21	41	68
**Yersinia infection**	hsa05135	Infectious disease: bacterial	11	21	42	124

Supplementary Table 3 - Average disease pathway similarity within KEGG subclasses. Listed is the average similarity, in terms of Jaccard index, between all disease pathways within the same KEGG subclass, for disease pathways in the Disease Pathway Network using the top 20 linked pathways.

KEGG subclass	avg JI
**Cancer: specific types**	0.45
**Infectious disease: viral**	0.40
**Immune disease**	0.40
**Infectious disease: parasitic**	0.33
**Neurodegenerative disease**	0.32
**Substance dependence**	0.28
**Infectious disease: bacterial**	0.20
**Cardiovascular disease**	0.18
**Cancer: overview**	0.16
**Endocrine and metabolic disease**	0.08

Supplementary Table 4 - Summary of performances with top 20 links per target pathway in the disease pathway network. True Positive (TP), False Negative (FN), False Positive (FP), True Negative (TN), True Positive Rate (TPR), False Positive Rate (FPR), G-mean and 1 - relative rank are extracted over all predictions.

Method	TP	FN	FP	TN	1-Rank	TPR	FPR	G-mean
**ANUBIX-FunCoup**	611	1118	3526	48344	0.60	0.35	0.07	0.57
**ANUBIX-STRING**	610	1119	3720	48150	0.62	0.35	0.07	0.57
**BinoX-FunCoup**	811	918	10546	41324	0.61	0.47	0.20	0.61
**BinoX-STRING**	977	752	16665	35205	0.65	**0.57**	0.32	**0.62**
**CAMERA**	489	1240	463	51407	0.48	0.28	0.01	0.53
**CAMERA***	177	1552	662	51208	0.51	0.10	0.01	0.32
**CePa-ORA**	349	1380	4971	46899	0.60	0.20	0.10	0.43
**EASE**	145	1584	525	51345	0.57	0.08	0.01	0.29
**fGSEA**	684	1045	2616	49254	0.52	0.40	0.05	0.61
**Fisher**	190	1539	1584	50286	0.53	0.11	0.03	0.33
**GSA**	187	1542	2179	49691	0.53	0.11	0.04	0.32
**GSEA**	684	1045	2631	49239	0.53	0.40	0.05	0.61
**GSVA**	680	1049	13104	38766	0.51	0.39	0.25	0.54
**NEAT-FunCoup**	775	954	10046	41824	0.60	0.45	0.19	0.60
**NEAT-STRING**	950	779	15241	36629	0.64	0.55	0.29	**0.62**
**netPEA-FunCoup**	560	1169	6320	45550	0.58	0.32	0.12	0.53
**netPEA-STRING**	621	1108	5635	46235	0.65	0.36	0.11	0.57
**PADOG**	188	1541	3051	48819	0.57	0.11	0.06	0.32
**ROAST**	738	991	18499	33371	0.51	0.43	**0.36**	0.52
**SPIA**	206	1523	1709	50161	**0.68**	0.12	0.03	0.34

Supplementary Table 5 - Spearman correlation *p*-values between pathway network properties and false positive rate. The correlation *p*-values were estimated with a test for correlation between paired samples for a selection of network properties against the False Positive Rate (FPR) of KEGG pathways as predicted by network-based EA methods with FunCoup and STRING as underlying functional association networks. Significant *p*-values below the cutoff 0.05 are marked in bold. See Supplementary Figure 5 to find the respective Spearman correlations.

	**FunCoup**				**STRING**			
	**ANUBIX**	**BinoX**	**NEAT**	**netPEA**	**ANUBIX**	**BinoX**	**NEAT**	**netPEA**
**Density of hubs**	9.6e-01	**1.6e-12**	**8.3e-07**	**1.5e-04**	**1.5e-09**	**1.6e-35**	**1.0e-28**	**2.5e-06**
**Density of links**	1.5e-01	**5.8e-10**	**3.1e-07**	1.2e-01	**6.4e-08**	**1.3e-02**	9.1e-02	2.1e-01
**Fraction of hubs**	**3.6e-02**	**2.1e-45**	**2.3e-32**	**2.9e-07**	**6.1e-18**	**2.2e-74**	**9.5e-60**	**7.8e-07**
**Fraction of intralinks**	2.8e-01	**5.8e-41**	**1.7e-50**	9.7e-01	**4.6e-10**	**1.8e-03**	**3.1e-06**	**2.2e-04**
**Max degree**	6.3e-01	**7.2e-09**	**9.2e-05**	**3.8e-04**	**6.7e-30**	**3.7e-39**	**3.1e-29**	**2.8e-15**
**Mean clustering coefficient**	**2.5e-04**	**6.8e-04**	**1.4e-02**	8.4e-01	**1.2e-12**	**6.9e-07**	**3.0e-05**	**3.2e-05**
**Median degree**	3.6e-01	**3.2e-19**	**2.1e-13**	**2.3e-06**	**1.2e-10**	**2.4e-38**	**6.9e-31**	**4.3e-07**
**Number of CC**	7.3e-01	8.6e-01	5.7e-01	3.9e-01	1.5e-01	9.8e-02	5.8e-02	5.2e-02
**Size**	**1.7e-02**	**8.6e-36**	**1.3e-33**	**8.5e-04**	**2.1e-15**	**7.6e-55**	**4.1e-47**	**3.2e-04**

References

1. Wang JZ, Du Z, Payattakool R, et al. A new method to measure the semantic similarity of GO terms. Bioinformatics 2007; 23:1274–1281
2. Goeman JJ, Bühlmann P. Analyzing gene expression data in terms of gene sets: methodological issues. Bioinformatics 2007; 23:980–987
3. Krämer A, Green J, Pollard J Jr, et al. Causal analysis approaches in Ingenuity Pathway Analysis. Bioinformatics 2014; 30:523–530
4. Hosack DA, Dennis G Jr, Sherman BT, et al. Identifying biological themes within lists of genes with EASE. Genome Biol. 2003; 4:R70
5. Huang DW, Sherman BT, Lempicki RA. Systematic and integrative analysis of large gene lists using DAVID bioinformatics resources. Nat. Protoc. 2009; 4:44–57
6. Subramanian A, Tamayo P, Mootha VK, et al. Gene set enrichment analysis: a knowledge-based approach for interpreting genome-wide expression profiles. Proc. Natl. Acad. Sci. U. S. A. 2005; 102:15545–15550
7. Korotkevich G, Sukhov V, Budin N, et al. Fast gene set enrichment analysis. bioRxiv 2016;
8. Efron B, Tibshirani R. On testing the significance of sets of genes. Ann. Appl. Stat. 2007; 1:107–129
9. Wu D, Lim E, Vaillant F, et al. ROAST: rotation gene set tests for complex microarray experiments. Bioinformatics 2010; 26:2176–2182
10. Hänzelmann S, Castelo R, Guinney J. GSVA: gene set variation analysis for microarray and RNA-seq data. BMC Bioinformatics 2013; 14:7
11. Wu D, Smyth GK. Camera: a competitive gene set test accounting for inter-gene correlation. Nucleic Acids Res. 2012; 40:e133
12. Tarca AL, Draghici S, Bhatti G, et al. Down-weighting overlapping genes improves gene set analysis. BMC Bioinformatics 2012; 13:136
13. Tarca AL, Draghici S, Khatri P, et al. A novel signaling pathway impact analysis. Bioinformatics 2009; 25:75–82
14. Gu Z, Wang J. CePa: an R package for finding significant pathways weighted by multiple network centralities. Bioinformatics 2013; 29:658–660
15. Nguyen T-M, Shafi A, Nguyen T, et al. Identifying significantly impacted pathways: a comprehensive review and assessment. Genome Biol. 2019; 20:1–15
16. Liu L, Ruan J. Network-based Pathway Enrichment Analysis. Proceedings Proceedings (IEEE Int Conf Bioinformatics Biomed) 2013; 218–221
17. Signorelli M, Vinciotti V, Wit EC. NEAT: an efficient network enrichment analysis test. BMC Bioinformatics 2016; 17:352
18. Ogris C, Guala D, Helleday T, et al. A novel method for crosstalk analysis of biological networks: improving accuracy of pathway annotation. Nucleic Acids Res. 2017; 45:e8
19. Castresana-Aguirre M, Sonnhammer ELL. Pathway-specific model estimation for improved pathway annotation by network crosstalk. Sci. Rep. 2020; 10:13585
